# Short-Term Efficacy of Rofecoxib and Diclofenac in Acute Shoulder Pain: A Placebo-Controlled Randomized Trial

**DOI:** 10.1371/journal.pctr.0020009

**Published:** 2007-03-09

**Authors:** Maxime Dougados, Anne Le Henanff, Isabelle Logeart, Philippe Ravaud

**Affiliations:** 1 Faculté de Médecine, Université Paris-Descartes, Paris, France; 2 Service de Rheumatologie, Assistance Publique-Hôpitaux de Paris, Hôpital Cochin, Paris, France; 3 Institut National de la Santé et de la Recherche Médicale (INSERM) E0357, Faculté Xavier Bichat (Université Paris 7), Paris, France; 4 Département d'Epidémiologie, Biostatistique et Recherche Clinique, Groupe Hospitalier Bichat-Claude Bernard (Assistance Publique–Hôpitaux de Paris), Paris, France; 5 Merck, Sharp and Dohme Chibret Laboratories, Paris, France

## Abstract

**Objectives:**

To evaluate the short-term symptomatic efficacy of rofecoxib and diclofenac versus placebo in acute episodes of shoulder pain.

**Design:**

Randomized controlled trial of 7 days.

**Setting:**

Rheumatologists and/or general practitioners totaling 47.

**Participants:**

Acute shoulder pain.

**Interventions:**

Rofecoxib 50 mg once daily, diclofenac 50 mg three times daily, and placebo.

**Outcome measures:**

Pain, functional impairment, patient's global assessment of his/her disease activity, and local steroid injection requirement for persistent pain. The primary variable was the Kaplan-Meier estimates of the percentage of patients at day 7 fulfilling the definition of success (improvement in pain intensity and a low pain level sustained to the end of the 7 days of the study; log-rank test).

**Results:**

There was no difference in the baseline characteristics between the three groups (rofecoxib *n* = 88, placebo *n* = 94, and diclofenac *n* = 89). At day 7, the Kaplan-Meier estimates of successful patients was higher in the treatment groups than in the placebo (54%, 56%, and 38% in the diclofenac, rofecoxib, and placebo groups respectively, *p* = 0.0070 and *p* = 0.0239 for placebo versus rofecoxib and diclofenac, respectively). During the 7 days of the study, there was a statistically significant difference between placebo and both active arms (rofecoxib and diclofenac) in all the evaluated outcome measures A local steroid injection had to be performed in 33 (35%) and 19 (22%) patients in the placebo and rofecoxib group respectively. Number needed to treat to avoid such rescue therapy was 7 patients (95% confidence interval 5–15).

**Conclusion:**

This study highlights the methodological aspects of clinical trials, e.g., eligibility criteria and outcome measures, in acute painful conditions. The data also establish that diclofenac and rofecoxib are effective therapies for the management of acute painful shoulder and that they reduce the requirement for local steroid injection.

## Editorial Commentary

**Background:** Shoulder pain is a very common complaint that presents in primary care, and there are many different possible causes. Acute pain would normally be managed with nonsteroidal anti-inflammatory drugs (NSAIDs), supplemented with steroid injections (which are often reserved for the treatment of severe or persistent pain). One NSAID, diclofenac, is used frequently for this condition, but other NSAIDs might also be effective. A subgroup of NSAIDs called the Cox-2 selective inhibitors specifically inhibit one particular enzyme (cyclo-oxygenase, shortened to Cox-2) which is involved in inflammation and pain. These drugs are thought to be less likely to cause stomach irritation than other NSAIDs. Therefore the researchers in this study carried out a short-term, three-way clinical trial comparing diclofenac with one particular Cox-2 inhibitor, rofecoxib, and placebo in patients with acute shoulder pain. However, rofecoxib was withdrawn from the market in September 2004 because of evidence that use of the drug was associated with an increased risk of heart attacks and strokes, and controversy remains regarding the risk of such events among users of other Cox-2 inhibitors.

**What this trial shows:** The main aim of this trial was to compare the level of pain relief over seven days of treatment with either diclofenac or rofecoxib, as compared to placebo. The primary outcome measure used in the trial was the proportion of patients achieving a 50% or greater decrease in pain levels over the course of the study, measured using a numerical rating scale. A total of 273 participants were recruited into the trial and at day 7 the proportion achieving a 30% decrease in pain was 38% in the placebo arm, 54% in the diclofenac arm, and 56% in the rofecoxib arm. The differences in this outcome measure between diclofenac and placebo and between rofecoxib and placebo were statistically significant; however, the researchers did not carry out a direct comparison between diclofenac and rofecoxib. The rates of adverse events were roughly comparable between all three arms of the trial, although the study was not originally planned to be large enough to detect differences in the rates of such events, so it is not possible to conclude whether there was any true difference.

**Strengths and limitations:** The randomization procedures used in the study minimize the possibility of bias in assigning patients to treatment arms. Bias in assessment of outcomes was also minimized by ensuring that steps were taken to prevent investigators and patients from knowing which drugs a particular patient received until the end of the trial. A key limitation of the study is the short follow-up, only seven days, and it is therefore unclear whether efficacy and safety of these drugs would continue for the much longer periods of time (weeks or even months) for which these patients might need pain relief. Finally, patients randomized to the placebo arm received no treatment for the seven days of the study other than acetaminophen or steroid injections (which would result in withdrawal from the trial). This design does not limit interpretation of the data but could be criticized because of concern over whether the patients receiving placebo received adequate pain relief.

**Contribution to the evidence:** This study provides some data on the efficacy of diclofenac and rofecoxib, as compared to placebo in treatment of this condition. Given that rofecoxib is now withdrawn, the efficacy of this drug is no longer relevant. However, the information from this trial should help in designing future studies of NSAIDs in shoulder pain, for example to define appropriate trial outcomes, sample size, and other aspects of study design.

## INTRODUCTION

Shoulder pain is the third most common musculoskeletal symptom encountered in medical practice after back and neck pain [1,2]. A wide range of potential pathoanatomic entities can give rise to shoulder pain, from simple sprains to massive rotator tears [3]. Most of these different conditions can be adequately diagnosed after careful interview and physical examination of the patient [4]. Options for the medical management of acute shoulder pain include systemic (e.g., nonsteroidal anti-inflammatory drugs [NSAIDs]) and local (e.g., injection of steroids) approaches. Several studies have investigated the efficacy of injectable steroids and/or the combination of injectable steroids and NSAIDs [5–12].

Given the invasiveness of injections, many practitioners prefer an initial trial of oral NSAIDs with injections reserved for patients with persistent pain or severe pain at the time of initial presentation [3]. Despite this pragmatic approach, such a procedure was until recently [13] little evaluated. Among the different conventional NSAIDs, diclofenac is one of the most frequently used for such conditions in daily practice [14]. Because of a better gastrointestinal safety profile, coxibs might be preferred in particular in patients at risk of gastrointestinal complications [15]. Efficacy of rofecoxib 50 mg once daily has been demonstrated in different acute pain human models (e.g., post–dental surgery [16], primary dysmenorrhea [17], and post–orthopedic surgery pain [18]).

These findings prompted us to conduct a seven-day clinical trial evaluating the short-term symptomatic efficacy of rofecoxib in acute, painful episodes of rotator-cuff syndrome using both potentially negative (placebo) and active (diclofenac) control groups. A secondary but clinically relevant end point was the requirement for local steroid injections. Although one of the study drugs (rofecoxib) was withdrawn from the market in October 2004, the data are being reported now for the sake of completeness and transparency.

## METHODS

### Participants

The inclusion criteria were selected to optimize the probability of recruiting patients suffering from an acute painful episode of tendonitis of the rotator cuff. For this purpose, the following criteria were chosen: patients less than 60 years of age, suffering from an acute (less than 7 d), painful (numerical rating scale [NRS] 0–10 ≥ 5 in which 0 and 10 are the best and worst conditions, respectively), episode occurring in the shoulder area with the following findings at physical examination: no limitation of the glenohumeral external rotation and a painful Job test [19,20]. A schema explaining the Job test was provided to all the 47 investigators. Moreover, in order to enter the trial, the patients had to have no contraindication for receiving either diclofenac or rofecoxib (e.g., history of NSAID allergy; renal, liver, or cardiac failure; or pregnancy).

Any NSAID intake other than the study drug was prohibited during the study. In case of previous intake, the NSAIDs had to be discontinued at least 3 d before enrollment. During the baseline visit, the following information was also collected: lateralization (right- or left-handedness) and history of progression of the symptoms with the a priori defined following four categories: (i) no history of chronic pain before this episode; (ii) history of chronic pain before this first acute episode; (iii) history of similar acute episodes without any history of chronic pain between episodes; and (iv) history of similar acute episodes with history of chronic pain between episodes. Therefore, the date of onset of shoulder disease was not always that of the current episode.

### Study Design

The study was a double-blind, 7-d randomized controlled trial comparing placebo, rofecoxib 50 mg, and diclofenac 150 mg using a double-dummy technique. The design of the study was approved by the ethics committee of Cochin Hospital (Paris, France). All patients gave their written informed consent before entering the trial. The study was conducted in 47 centers from 15 April 2003 to 16 March 16 2004.

### Interventions

#### Study drugs.

After confirmation of patient eligibility and after written informed consent was obtained, patients were randomly assigned to receive placebo, diclofenac 50 mg three times daily, or rofecoxib 50 mg once daily. All the patients took four capsules per day (two at breakfast, one at lunch, and one at dinner) during the seven days of the trial, regardless of the level of symptoms and the randomization group. Capsules and packages were identical in appearance.

Compliance was evaluated by pill count at the final visit.

#### Rescue therapies.

Acetaminophen (500 mg tablets, maximum eight tablets/day) was used as analgesic treatment during the study when needed. Since acetaminophen was supplied as part of the study, a pill count of acetaminophen was performed at final visit.

In cases of persistent intolerable pain, a local injection of steroids was performed. This injection was defined as treatment failure, resulting in the withdrawal of the patient from the trial.

### Objectives

The objective of this trial was to demonstrate the superiority of NSAIDs over placebo in acute shoulder pain over a 7-d treatment period.

### Outcomes

#### Primary outcome measure.

Changes in pain were considered as the primary outcome measure. The original trial protocol was referring to a “clinically relevant” definition of the primary outcome (e.g., a success defined by a sustained improvement of at least 50% and an absolute level of pain of 30 or less [0–100 normalized scale]). While the recruitment of patients was still ongoing and based on discussion concerning the potential loss of statistical power by using a dichotomous variable instead of a continuous variable, an amendment was proposed and accepted by the ethical committee to redefine the primary variable as the mean changes in pain during the study. Since both techniques resulted in similar findings, we are presenting here the results according to the original trial protocol. For this purpose, a diary was provided to the patient in order to collect twice a day (in the morning and in the evening) his/her pain intensity over the 12 previous hours (“nocturnal” pain collected in the morning and “diurnal” pain collected in the evening) using a 0–10 NRS.

#### Secondary outcome measures.

Functional impairment and patient's global assessment were considered as secondary symptomatic outcome measures. Clinical assessment was performed at baseline and after 7 d by the same investigator, and functional impairment and patient's global assessment were collected. Functional impairment was evaluated using the function subscale of Neer's index [21]. This scale consists of ten questions related to daily activities (1, use back pocket; 2, perineal care; 3, wash opposite axilla; 4, eat with utensil; 5, comb hair; 6, use hand with arm at shoulder level; 7, carry 10–15 pounds with arm at side; 8, dress; 9, sleep on side; 10, do usual work). For each question, a score of 0 was assigned if the activity could be performed without any difficulty, 1 with some difficulty, 2 with marked difficulty, 3 with great difficulty (requiring assistance), and 4 if impossible. Therefore, this scale ranges from 0 to 40.

Patients' global assessments were evaluated using three different techniques. In one, at the baseline and the final visits, patient's global assessment of disease activity was collected by the following question “considering all the ways your shoulder disease affects you, mark an (X) in the appropriate box for how well you are doing” and the following potential answers 0, very well; 1, well; 2, fair; 3, poor; and 4, very poor. For the analysis, this variable was considered as a continuous one and normalized from 0 = best condition to 100 = worst condition.

In a second technique, at the final visit, patient's global assessment on her/his relative condition was collected by the following question: “compared to when you started the study, how have you been during the last 48 hours?” and the following potential 15 answers from −7, very great deal worse to +7, very great deal better. For the analysis, we considered this variable as a dichotomous one (e.g., improvement yes/no in which an improvement was considered for the patients answering at least “good deal better”).

In a third technique, at the final visit, acceptable symptom state was assessed by the following question: “considering your current level of pain and functional impairment, if you were to remain for the next following months as you were during the last 48 hours would this be acceptable or unacceptable to you?” and the potential following answers 0, acceptable; 1, not acceptable.

Moreover, the requirement for rescue therapies (e.g., acetaminophen intake and/or local injection of steroids) was also considered as a secondary outcome measure of efficacy.

At baseline and final visit, blood pressure and body weight were systematically collected and at final visit, the investigators checked for tolerability.

### Sample Size

The sample size needed in order to demonstrate a statistically significant difference between NSAID and placebo using the pain intensity (mean of nocturnal and diurnal pain) was calculated. A success was a priori defined by a sustained improvement of at least 50% and an absolute level of pain 30 or less (on a 0–100 normalized scale). The analysis was conducted using the Kaplan-Meier technique in which the event was defined by the time the patient fulfilled the above definition of improvement in pain with such an improvement sustained until the end of the study. Based on information on changes in pain in previously reported trials [22], the Kaplan-Meier estimates of the percentage of patients achieving such an improvement was expected to be around 40% in the active group. Since, to our knowledge, no information was available in the literature concerning the placebo group, we a priori and arbitrarily expected a 20% success rate in the placebo group. Thus a sample size of 82 patients per treatment group would allow demonstration of this difference with an alpha level of 0.05 and power of 0.80 two-tailed).

### Randomization: Sequence Generation

A computer-generated randomization sequence assigned participants in a 1:1:1 ratio to receive rofecoxib, diclofenac, or placebo using a block size of six. For each individual patient recruited in the trial, a coded package for the drugs was used.

### Randomization: Allocation Concealment

The randomization code was available only to the statistician who did not participate in any way for patient recruitment. The code was revealed to the researchers once recruitment, data collection, and statistical analysis were complete. Because of the double-dummy technique used with matching placebo, allocation was concealed from both investigators and patients.

### Randomization: Implementation

After checking that screened patients fulfilled the inclusion/exclusion criteria of the study and after written informed consent was obtained, the investigator assigned the patient on an individual basis to an allocated number. The patient remained on the same allocation throughout the study. The investigators responsible for seeing the patients allocated them to the next available number into the trial (in the rheumatologist/general practitioner's office), and provided the treatment corresponding to the allocated number of the patients.

### Blinding

This was a double-blind trial. Both the patients and the physicians providing care were blinded of the study drug allocation. For this purpose, a coded package for the drugs was used. The active drugs and their corresponding placebos were identical in appearance, color, and taste. The blinding process remained complete until the database (e.g., all the information concerning the patients and the course of the disease during the seven days of the study) was completed and was locked.

### Statistical Methods

The efficacy analysis was conducted on the modified intention to treat population, defined as all patients randomized in the study and receiving at least one dose of study drug with the last observation carried forward technique. For patients who withdrew without assessment, the baseline value was reported as the final value. All patients were analyzed in the group to which they were randomized.

The primary efficacy variable (success of treatment was defined by a decrease in pain of at least 50% and an absolute level ≤30) was evaluated using life table analysis (Kaplan-Meier technique) in which the event was defined by the time to reach a sustained definition of success until the end of the study. The results are expressed as Kaplan-Meier estimates of the percentage of success per treatment group at day 7. The primary analysis consisted in the comparison between rofecoxib and placebo groups using the log-rank test. As a secondary analysis, diclofenac was also compared to placebo according to the same definition of success.

Exploratory analyses were also performed using the same approach (life table analysis) but with different definitions of event (success): time to reach an improvement of 50%; time to reach an improvement of 50% and such an improvement is maintained during the remaining days of the study; time to reach an absolute level of pain ≤ 30; and time to reach an absolute level of pain ≤ 30 and such a good condition is maintained during the remaining days of the study.

The continuous secondary variables are also expressed in normalized units using a 0–100 scale in which 0 = best condition and 100 = worst condition. Absolute changes between day 0 and day 7 of “nocturnal pain,” diurnal pain,” “number of pills of acetaminophen,” “functional impairment,” and “patient's global assessment” were compared with a nonparametric Mann-Whitney test. The dichotomous secondary variables (“patients considering their condition as improved,” “patients considering themselves as feeling well,” “patients considering their current status as acceptable,” and “patients requiring a local injection of steroids”) were compared using the Chi-square test.

In order to estimate the clinical relevance of the observed treatment effect on the requirement for local steroid injection, the number needed to treat and its 95% confidence interval were calculated.

Statistical analysis was performed using SAS version 8.2 (SAS Institute, http://www.sas.com).

## RESULTS

### Participant Flow

**Figure 1:**
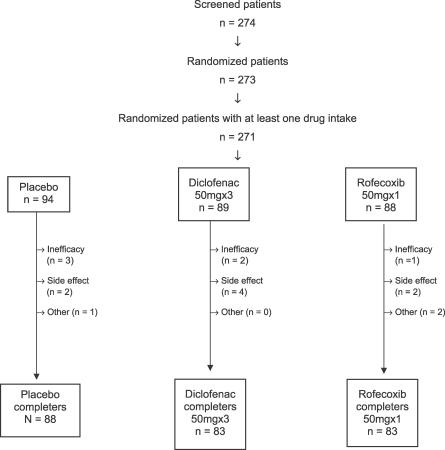
Patients and Study Course

Of the 274 screened patients, 273 were randomized but only 271 had at least one drug intake (see Figure 1). Most of the patients completed the 7 d of the trial (93.6%, 93.3%, and 94.3% completer rate in the placebo, diclofenac, and rofecoxib groups, respectively). The main reason for discontinuation was either inefficacy (3, 2, and 1 patients in the placebo, diclofenac, and rofecoxib groups, respectively) or side effects (4, 2, and 2 patients in the placebo, diclofenac, and rofecoxib group, respectively).

Compliance was considered as good since the mean capsule intake was 94.5%, 93.0%, and 93.8% of the theoretical total in the placebo, diclofenac, and rofecoxib groups, respectively.

### Recruitment Period

The recruitment of the patients started in April 2003 and ended in March 2004.

### Baseline Data

Table 1 summarizes patient characteristics at the start of the trial by treatment group. There was no obvious difference in demographic data or clinical variables among the three treatment groups at baseline.

**Table 1:**
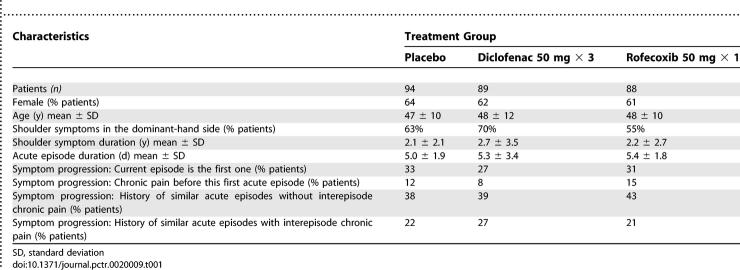
Baseline Characteristics of the 271 Randomized and Treated Patients with Painful Rotator Cuff Syndrome by Treatment Group

### Number Analyzed

All the randomized patients with at least one drug intake were analyzed (i.e., 94, 89, and 88 in the placebo, diclofenac, and rofecoxib groups, respectively)

### Outcomes and Estimation

**Figure 2:**
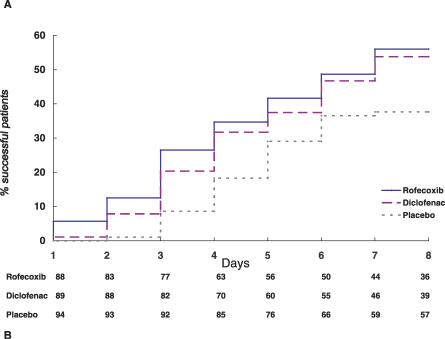
Percentage of Acute Painful Shoulder Patients Fulfilling the Definition of Success over Time by Treatment Group (A) Success was defined by a decrease in pain of at least 50% and an absolute level ≤ 30 (on a 0–100 scale). Moreover, such definition required that such condition should be sustained until the end of the 7 days of the study. (B) Number of patients at risk per treatment group at the beginning of each interval.

The percentage of patients achieving the definition of success over time during the trial by treatment group is summarized in Figure 2. The Kaplan-Meier estimates of the percentage of success at day 7 was 38%, 54%, 56% in placebo, diclofenac, and rofecoxib groups, respectively, with a highly significant difference between the placebo and rofecoxib groups (*p* = 0.025). Table 2 shows the percentage of patients considered as a success by treatment group at the end of the trial considering not only the primary efficacy criterion (sustained improvement of at least 50% and absolute level of pain ≤30 on a 0–100 scale) but also the secondary efficacy variables concerning the definition of success. Similar results were obtained when success was defined as pain lessening using the same analysis (life table analysis) but with different thresholds to define the event (unpublished data).

**Table 2:**
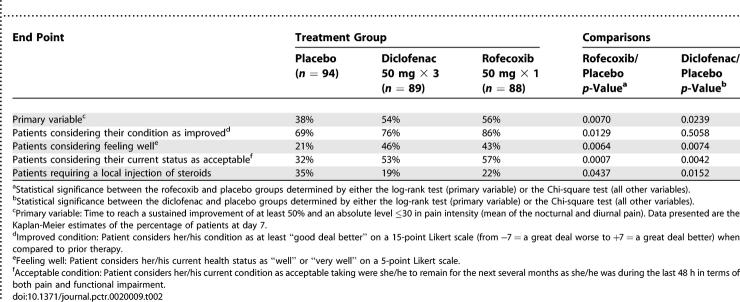
Percentage of Patients Considered as Treatment Success Based on Different Definitions by Treatment Group at the End of the Study

The mean changes in the different continuous variables by treatment groups are summarized in Table 3. Except for acetaminophen pill intake, all the variables strongly favored the rofecoxib and/or the diclofenac group when compared to placebo.

**Table 3:**
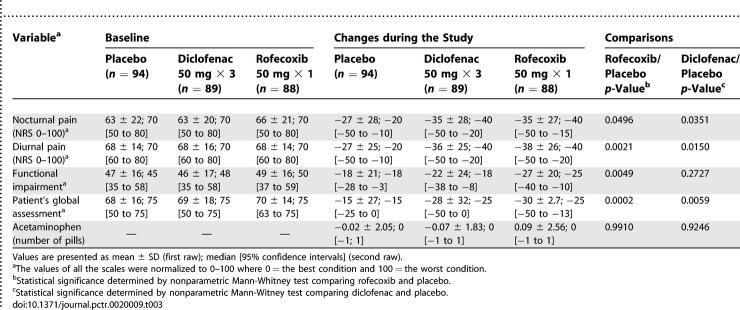
Baseline Values and Mean Changes in Clinical Variable and Use of Acetaminophen after Seven Days of the Study, by Treatment Group

At the end of the trial, the percentage of patients considering their condition as substantially improved or as acceptable was more important in the two active groups than in the placebo group (see Table 2). Moreover, during the seven days of the study (and most often at the final visit), a local injection of steroids was required by 35% of the patients receiving placebo, a higher percentage than in the diclofenac and rofecoxib groups (19% and 22%, respectively). Based on these data, it was estimated that the number of patients needed to treat in order to avoid the requirement of one local injection of steroids (number needed to treat) was 7 (95% confidence interval 5–15).

### Adverse Events

Thirty-nine patients (13 patients in each group) experienced at least one adverse event (53 in total) during the study. Gastrointestinal disorders (mainly upper abdominal pain and nausea) occurred in 12 (12.8%), 11 (12.4%), and 9 (10.2%) in the placebo, diclofenac, and rofecoxib groups, respectively.

In the rofecoxib group, one patient had to be hospitalized 2 d after the end of the study because of an ulcer perforation; outcome was favorable after surgery. There was no death and no cardiovascular event. During the study, eight patients had to discontinue the study drug mainly because of gastro-intestinal discomforts. The most frequent adverse events observed during the trial are summarized in Table 4.

**Table 4:**
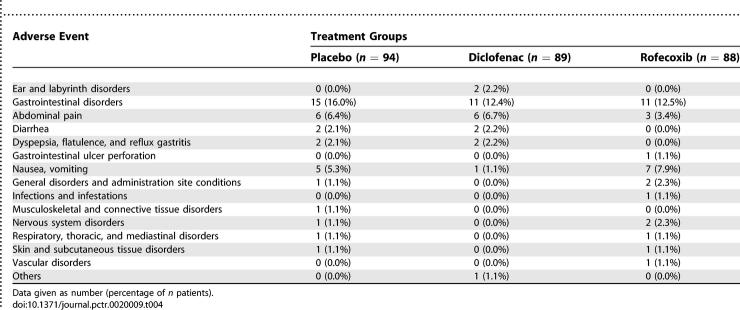
Adverse Events by Treatment Group Observed during the Seven Days of the Trial

## DISCUSSION

### Interpretation

This study confirms the short term symptomatic efficacy of NSAIDs (either conventional such as diclofenac or selective Cox-2 inhibitors such as rofecoxib) in acute shoulder pain. The observed treatment effect in the a priori selected outcome variable (i.e., 54% and 56% versus 38% in the diclofenac and rofecoxib versus placebo groups) reached statistical significance and was also close to the a priori expected treatment effect (i.e., a difference of 20% between active and placebo). The interpretation of results is made difficult by the fact that there is no clear consensus on the primary outcome variable which should be chosen in this type of study. However, the positive and statistically significant results in favor of NSAIDs in the secondary outcome variables, especially the requirement for steroid injections, support the primary analysis. Finally, the interpretation of the results should also take into account issues related to both the eligibility criteria, the outcome measures, and the choice of the study drugs (in particular the placebo).

### Eligibility Criteria

There is no current, formal recommendation for conducting clinical trials in shoulder pain syndrome, at least to our knowledge. The design of the currently reported trial was based on knowledge of the disease and of the commonly used outcome variables in musculoskeletal disorders. Concerning the disease, reviews on diagnostic criteria for shoulder pain conclude that current diagnostic tools are not evidence based [23]. The main objective when establishing exclusion criteria of the present study were to try to avoid the inclusion of patients suffering from glenohumeral osteoarthritis and/or adhesive capsulitis. For this purpose, we excluded patients over 60 years old and patients with an abnormal glenohumeral external rotation (the most commonly limited range of motion in osteoarthritis and/or adhesive capsulitis [4]). The second objective was to try to avoid patients with massive rotator cuff tears and/or ruptures. Based on the data found in the literature, we considered that the Job's test was the most discriminant for this purpose [19,20].

### Outcome Measures

While designing the trial, concerning the choice of the outcome measures used in this trial, we considered three key aspects: Which domain? Which instrument? Which analysis? The ultimate goal of any therapeutic intervention for shoulder pain is the restoration of pain-free function [3]. Based on this objective, we focused our research on the three domains commonly selected in musculoskeletal disorders, i.e., pain, functional impairment, and the patient's global assessment. For each of these domains, we selected specific tools, items, and/or instruments we considered to be the most adequate. For the domain “pain,” it seemed important to collect its degree of severity both during the night and during daily activities. There is no consensus related to the optimal questionnaire evaluating shoulder disability [24]. The scoring system proposed by Neer is a composite index combining the information from four different domains: pain, range of motion, functional impairment, and patient's global [21]. This scoring system is frequently used for the evaluation of orthopedic procedures. For our trial we considered that the function subscale was appropriate, since it is relevant and simple to use. Finally patient's global assessment is an important domain to consider, as was recently re-emphasized during a meeting about rheumatology and methodology for trials [25]. In our study, the patient's global assessment was evaluated in different ways. First, we used a “conventional” approach by evaluating the changes during the study of the current status of the patient using a 5-point Likert scale [26,27]. Second, we evaluated the concept of minimum clinically important improvement [28] by questioning the patient at the end of the study about the clinical relevance of the changes she/he noted during the study [29]. Thirdly, we also evaluated the concept of patient-acceptable symptom state by questioning the patient at the end of the trial about her/his acceptability of the current status [30].

The report results also anticipated considering that presentation at an individual level is probably more relevant than presentation at a group level [28–30]. Therefore, instead of choosing mean changes as the primary variable, we selected the presentation at an individual level. There are several possibilities of presentation of results at an individual level: the first step is to choose between the concept of responder (to be in a better condition) and the concept of status (to be in a good condition). The concept of status seemed to be more relevant [31] and more appropriate in acute painful conditions in which the main objective is to attain a pain-free condition [3]. Moreover, the onset of action is also important to consider. This concept (to feel good as soon as possible and to maintain such a good condition during the study) was the rationale of the choice of the primary variable used in this study.

The choice of the threshold of 30/100 in degree of severity of pain below which the patient considers her/his status as acceptable was arbitrarily chosen but was close to other patient-acceptable symptom state thresholds recently reported [30,32].

### Study Drugs (Placebo Arm)

Because of the huge variability of symptoms in most painful musculoskeletal disorders, a placebo arm is usually encouraged in order to facilitate the interpretation of the results. Such a placebo arm seems acceptable from an ethical point of view if the patient has the opportunity to obtain an optimal rescue therapy. This was the case in this trial during which a persistent painful condition was treated by use of acetaminophen and if necessary by local injections of steroids.

Despite the fact that we did not perform formal comparative statistical testing, data obtained in this trial are in favor of a similar treatment effect for rofecoxib and diclofenac. However, we have to re-emphasize the fact that because of cardiovascular safety concerns, rofecoxib has been withdrawn from the market. This study, conducted during a very short period of time without further follow-up of patients, cannot contribute toward the assessment of safety.

### Generalizability

Based on this study, it is hard to generalize the obtained results with regard to both the study drugs and clinical condition being evaluated. In particular, such a study does not allow a conclusion that any NSAID at any dose might be beneficial in such conditions. Also, such a study does not allow conclusions to be made that positive clinical benefit may be identical in another musculoskeletal condition (such as epicondylitis or achilleus tendinitis).

### Overall Evidence

A Cochrane review on therapeutic interventions for acute shoulder pain currently does not include an estimate of the treatment effect of NSAIDs for this particular condition [33]. A recent systematic literature review found inconclusive evidence on the effects of oral NSAIDs in people with shoulder pain [34]. Positive results with other NSAIDs/coxibs have been reported, but only in open and/or comparative trials [5,8,21,35]. A single trial, using a prospective placebo design (similar to the currently reported one), compared the two-week symptomatic efficacy of celecoxib 200 mg twice daily and naproxen 500 mg twice daily versus placebo, and showed similar results with a superiority of both celecoxib and naproxen when compared to placebo [13].

Finally, other studies are needed in order to evaluate the best dose regimen and the best therapeutical strategy, in particular whether it might be more clinically relevant to perform as the first step an NSAID/coxib intake and thereafter (in case of failure) a local injection of steroids (as has been evaluated in this trial) versus a local injection of steroids without oral drugs as the first step versus the combination of oral drug and local injection of steroids.

## SUPPORTING INFORMATION

CONSORT Checklist Click here for additional data file. (50 KB DOC)

Trial Protocol (Original) Click here for additional data file. (413 KB DOC)

Trial Protocol (Amendment) Click here for additional data file. (384 KB DOC)

## Abbreviations

NNT: number needed to treat
NRS: numerical rating scale
NSAID: nonsteroidal anti-inflammatory drug
