# Correction: Short-Term Efficacy of Rofecoxib and Diclofenac in Acute Shoulder Pain: A Placebo-Controlled Randomized Trial

**DOI:** 10.1371/journal.pctr.0020024

**Published:** 2007-05-04

In *PLoS Clinical Trials*, volume 2, issue 3, doi: 10.1371/journal.pctr.0020009:

The following competing interest was declared by the author on submission, but due to an error the text was not included in the published version of the article: “Philippe Ravaud has received travel grants from Merck Sharpe and Dohme for participation in meetings.”

