# Supplementary material for: Short-Term Efficacy of Rofecoxib and Diclofenac in Acute Shoulder Pain: A Placebo-Controlled Randomized Trial
Source: PLoS Clin Trials. 2007 Mar 9;2(3):e9. doi: 10.1371/journal.pctr.0020009 (PMC1817652; doi:10.1371/journal.pctr.0020009)
Supplement: Trial Protocol (Amendment) [file pctr.0020009.sd003.doc]

SPONSOR

Laboratoires MERCK SHARP & DOHME-CHIBRET

3, avenue Hoche

75114 PARIS Cedex 8

FRANCE

TITLE:

**Randomized, double-blind, 7-day study of the efficacy and safety of rofecoxib vs. placebo and diclofenac in patients with acute painful Rotator Cuff Syndrome.**

INVESTIGATOR:

**Professeur Maxime DOUGADOS**

**Service de Rhumatologie B**

**HOPITAL COCHIN-TARNIER**

**27, rue du Faubourg Saint-Jacques**

**75679 PARIS CEDEX 14**

**Tél. : 01-58-41-25-62**

SITE:

INSTITUTIONAL REVIEW BOARD/ETHICS REVIEW COMMITTEE:

### CCPPRB PARIS-COCHIN

## TABLE OF CONTENTS

**Page**

**I. CLINICAL SECTIONS 12**

**A. CONTEXT AND RATIONALE 12**

**1. Introduction 12**

**2. Value of selective COX-2 inhibitors 13**

**3. Rationale for Dose selection 13**

**B. OBJECTIVES 14**

**1. Primary objective 14**

**2. Secondary objectives 14**

**C. HYPOTHESES 14**

**D. PATIENT POPULATION DEFINITION 14**

**1. Inclusion criteria 14**

**2. Exclusion criteria 16**

**E. STUDY DESIGN 17**

**1. Summary of study design 17**

**2. Treatment 17**

**a. Treatment regimen 17**

**b. Prior and concomitant medication(s)/treatment(s) 18**

**c. Treatment compliance 18**

**3. Data collected during the study 18**

**a. Demographic data 18**

**b. Disease characteristics 18**

**c. Medical history 19**

**d. Concomitant medications 19**

**e. Efficacy criteria 19**

**f. Safety measurements 21**

**F. STUDY PROCEDURES 22**

**1. Visit 1 - Screening/Inclusion 22**

**2. Visit 2 - After 7 days of treatment 24**

**3. Study discontinuation visit 24**

**4. Unblinding procedure 25**

**5. Non compliance with treatment 25**

**G.** **DATA ANALYSIS** **26**

1. **Responsibility for Analyses/In-House Blinding 26**
2. **Variables analyzed 26**
3. **Risk of error 27**
4. **Hypothesis 27**
5. **Sample size calculation 27**
6. **Management of missing data 27**

**II. ADMINISTRATIVE AND REGULATORY SECTIONS 28**

1. **LABELING, PACKAGING, STORAGE, AND RETURN OF STUDY MEDICATIONS 28**
2. **Product Descriptions 28**
3. **Primary Packaging and Labeling Information 28**
4. **Secondary Packaging and Labeling Information (kit) 29**
5. **Standard procedures / Drug accountability / Comparator statement 30**
6. **CLINICAL DATA COLLECTION 30**
7. **STUDY DOCUMENTATION AND RECORDS RETENTION 31**
8. **INFORMED CONSENT 31**
9. **INSTITUTIONAL REVIEW BOARD (IRB)/INDEPENDENT ETHICS COMMITTEE (IEC) 32**
10. **CONFIDENTIALITY 32**
11. **COMPLIANCE WITH LAW, AUDIT, AND DEBARMENT 33**
12. **QUALITY CONTROL AND QUALITY ASSURANCE 33**
13. **PARTIE’S OBLIGATIONS 34**
14. **ADMINISTRATIVE ORGANIZATION OF THE TUDY 35**
15. **PUBLICATIONS 35**

**L. SIGNATURES 36**

**LIST OF REFERENCES 37**

**APPENDICES**

**1. Rofecoxib labeling 39**

**2. Diclofenac labeling 40**

**3. Jobe's test 41**

**4.Informed consent form 42**

**5. Patient information 43**

**6. Insurance certification 47**

## SYNOPSIS

**Study title:** Randomized, double-blind, 7-day study of the efficacy and safety of rofecoxib vs. placebo and diclofenac in patients with acute painful Rotator Cuff Syndrome.

**Primary criterion**: Comparing the short-term symptomatic efficacy (pain NRS) of rofecoxib (50 mg q.d.) versus placebo on mean changes in pain Numerical Rating Scale (NRS) (night pain + pain during physical activities/2) between D0 and D7.

**Study design**: Two visits are scheduled (inclusion and D7). The patients will receive rofecoxib or placebo or diclofenac and fill out a patient diary on daytime pain severity (NRS) during daily activities, night pain severity (NRS) and acetaminophen/paracetamol intake.

## INCLUSION CRITERIA

1. **Disease-related**

• Painful shoulder AND

• Painful Jobe's test but not positive and therefore not in favor of rotator cuff tear (cf. Appendix 3) AND

• No limitation of the external rotation of the shoulder (in order to eliminate advanced osteoarthritis and retractile capsulitis)

1. **Acute painful flare defined by:**

• Onset of the current episode less than 7 days prior the first visit AND

• Pain over the last 24 hours during daily activities greater than or equal to 5 on a NRS from 0 to 10

1. **Concomitant diseases at inclusion:**

Patients excluded if:

• NSAID intake in the last 72 hours

• Acetaminophen intake in the last 12 hours

- Local corticosteroid injection in the last 90 days.

**4. Patient age: less than 60 years** (in order to exclude osteoarthritic lesions)

**5. Other exclusion criteria:** refer to the case report form.

**STUDY MEDICATIONS :** The patients will receive rofecoxib 50 mg q.d or placebo or diclofenac 50 mg t.i.d using the double dummy technique

## CONCOMITANT MEDICATIONS DURING THE STUDY

1. NSAID are prohibited (in the event of a protocol violation, the patients will be considered failures in the statistical analysis)
2. Local injections of NSAID are prohibited (in the event of a protocol violation, the patients will be considered failures in the statistical analysis)
3. Acetaminophen/Paracetamol intake: the number of tablets taken will be reported in the patient's diary and checked by counting the tablets during visit 2. This parameter constitutes a secondary criterion for study treatment efficacy assessment.

**STUDY DURATION:** 7 days

## ASSESSMENT CRITERIA

1. **Pain during daily activities** assessed by a NRS at inclusion, then daily by the patient for 7 days. The last determination will be conducted on D7 in the presence of the investigating physician.
2. **Functional impairment** evaluated at inclusion and after 7 days of treatment using Neer's functional index with a 5-grade Likert scale.
3. Global assessment of **disease activity by the patient** using a NRS.
4. **Inflammation** defined by the intensity of night pain evaluated daily by NRS. A NRS determination will also be conducted at inclusion and on D7 in the presence of the investigating physician.
5. **Rescue treatment**: number of acetaminophen/paracetamol tablets taken by the patient during the 7 days of the study, requirement of a local corticosteroid injection in the shoulder during the 7 days of the study including the last visit at D7.
6. At the end of the study **Patient’s global assessment on her/his relative condition** when compared to baseline using a 15 grade scale in response to the following question:” ‘How do you assess your relative condition when compared to prior therapy”

**7.** At the end of the study **Patient’s global assessment on her/his current condition** using a binary (yes/no) variable in response to the following question: 'Taking into account all your daily activities and considering not only the pain but also your functional disability, do you consider that your current condition is satisfactory?'.

## STATISTICAL ANALYSIS

1. **Primary endpoints :**

The main analysis will evaluate the mean changes in pain (night pain + pain during physical activities/2) from D0 to D7. The first value will be recorded by the physician at baseline during the first visit. The following values will be recorded in the evening for pain NRS during the daily activities and the next morning for night pain. As a result, D0 will assess the pain recorded before treatment and D1 will assess the mean level of pain recorded the day prior to the study + the morning on the 1^st^ day of the study. The main effectiveness analysis will be a linear mixed model for repeated measures.

1. **Secondary endpoints :**

- Changes in pain during the physical activities (NRS)
- Changes in pain at night (NRS)
- Changes in functional index at D0 and D7
- Changes in patient’s global assessment of disease activity
- Patient’s global assessment of the response to the treatment at D7
- Patient’s global assessment of the level of state at D7
- Requirement of a local injection of corticosteroid in the shoulder during the 7 days of the study including the last visit at D7
- Number of acetaminophen/paracetamol tablets per day
- Tolerability

1. **Complementary statistical analyses:** the assessments indicated above will also be conducted on the diclofenac group with a descriptive aim.

## Sample SIZE CALCULATION

- We performed a literature analysis permitted us to detect a single study using a pain NRS at entry and after 7 days of NSAID therapy. (19):
- In this study, the reported value at inclusion for pain intensity (VAS) was 7 and the reported value on D7 was 40 mm with a standard deviation of 20 mm in the NSAID group. Thus, in the active treatment group, a 40% success rate is expected. No published data are available for the placebo group but, in the experts' opinion, a success rate of 20% may be expected.
- Log rank test
- two-tail testing
- *α* = 5%
- *β* = 20%
- On D7: expected placebo effect: 20%
- On D7: expected rofecoxib effect: 40%
- number of patients = 82 per arm
- Total number of patients
- Placebo = 82
- Rofecoxib = 82
- Proposed number of patients in the diclofenac group: n = 82

*ANY SERIOUS ADVERSE EXPERIENCE, INCLUDING DEATH DUE TO ANY CAUSE, WHICH OCCURS TO ANY PATIENT ENTERED INTO THIS STUDY OR WITHIN 28 DAYS FOLLOWING CESSATION OF TREATMENT OR WITHIN THE ESTABLISHED OFF-THERAPY FOLLOW-UP PERIOD FOR SAFETY DESCRIBED IN THE PROTOCOL, WHETHER OR NOT RELATED TO THE INVESTIGATIONAL PRODUCT, MUST BE REPORTED WITHIN 24 HOURS TO ONE OF THE INDIVIDUAL(S) LISTED ON THE CONTACT INFORMATION PAGE.*

*ADDITIONALLY, ANY SERIOUS ADVERSE EXPERIENCE CONSIDERED BY AN INVESTIGATOR WHO IS A QUALIFIED PHYSICIAN TO BE POSSIBLY, PROBABLY, OR DEFINITELY RELATED TO THE INVESTIGATIONAL PRODUCT THAT IS BROUGHT TO THE ATTENTION OF THE INVESTIGATOR AT ANY TIME OUTSIDE OF THE TIME PERIOD SPECIFIED IN THE PREVIOUS PARAGRAPH ALSO MUST BE REPORTED IMMEDIATELY TO ONE OF THE INDIVIDUALS LISTED ON THE SPONSOR CONTACT INFORMATION PAGE.*

**COMMENTARY**

**A. WHY ROTATOR CUFF SYNDROME?**

• Abarticular diseases are the most frequent musculo-squeletal disorders. They include diseases involving the following anatomical sites: tendons, ligaments, and articular capsule.

NSAIDs are usually considered as the standard pharmacological therapy in such conditions.

This therapeutic field has never been investigated with rofecoxib.

• Rotator Cuff Syndrome is a relevant model for such diseases since:

1. It is one of the most frequent tendinitis
2. The available diagnostic criteria (especially the Jobe’s Test) permit forming homogenous groups of patients
3. The available validated outcomes measures for the main domains (pain, functional impairment) allow the conduct of studies with real instrument

**B. WHICH PATIENTS?**

This study will address patients experiencing an **acute painful episode** related to tendinitis of the Rotator Cuff without tear.

In order to do so, two points require stressing:

• Inflammation criterion: patients presenting with intense pain (NRS ≥ 5) of recent onset

(< 7 days) will be included.

• Exclusion of other shoulder diseases such as retractile capsulitis and osteoarthritis: the clinical examination findings and exclusion of patients aged over 60 years will enable degenerative joint disease to be excluded.

**C. WHY THE TREATMENTS?**

• The placebo arm: a literature review has shown that, to the best of the authors' knowledge, there is no placebo-controlled study in the field. Conducting a placebo-controlled study therefore seems necessary in order to enable critical analysis of the results.

• The active comparator arm: diclofenac at a dosage of 50 mg t.i.d. is widely used in everyday practice. Numerous physicians consider diclofenac at that dosage a reference therapy with respect to the indication. The treatment arm will enable validation of that therapeutic model.

• A dosage of 50 mg rofecoxib q.d.: that dosage has been used successfully in controlled studies in clinical models of acute pain: dental pain, dysmenorrhea and post-operative pain. It is considered that the acutely painful syndrome related to Rotator Cuff Tendinitis constitutes an optimal approximation of the other clinical models of pain.

**D. WHY THE ASSESSMENT CRITERIA?**

Pain, functional impairment and patient’s global assessment are the three more important domains for the assessment of osteoarticular diseases. For Rotator Cuff disease, a functional index has been validated. The two other domains (pain and overall assessment by the patient) will be evaluated using standardized methods (NRS).

**E. WHY THE STATISTICAL ANALYSIS?**

Taking into account the lack of a validated index (PASS or MCII) to evaluate the success rate (global assessment by the patient of the response to the treatment and/or global assessment by the patient of his (her) current state at the end of the study) in the acute painful Rotator Cuff Syndrome and the fact that the continuous variable method is the only one to be accepted by the Registration Authorities, we suggest the main analysis should focus on the mean changes in pain *Numerical Rating Scale (night pain + pain during physical activities/2)* from D0 to D7.

# SPONSOR CONTACT INFORMATION

***REPORTING OF SERIOUS ADVERSE EXPERIENCES***

*ANY SERIOUS ADVERSE EXPERIENCE, INCLUDING DEATH DUE TO ANY CAUSE, WHICH OCCURS TO ANY PATIENT ENTERED INTO THIS STUDY OR WITHIN 14 DAYS FOLLOWING CESSATION OF TREATMENT OR WITHIN THE ESTABLISHED OFF-THERAPY FOLLOW-UP PERIOD FOR SAFETY DESCRIBED IN THE PROTOCOL, WHETHER OR NOT RELATED TO THE INVESTIGATIONAL PRODUCT, MUST BE REPORTED WITHIN 24 HOURS TO ONE OF THE INDIVIDUAL(S) LISTED ON THE CONTACT INFORMATION PAGE.*

*ADDITIONALLY, ANY SERIOUS ADVERSE EXPERIENCE CONSIDERED BY THE INVESTIGATOR WHO IS A QUALIFIED PHYSICIAN TO BE POSSIBLY, PROBABLY, OR DEFINITELY RELATED TO THE INVESTIGATIONAL PRODUCT THAT IS BROUGHT TO THE ATTENTION OF THE INVESTIGATOR AT ANY TIME OUTSIDE OF THE TIME PERIOD SPECIFIED IN THE PREVIOUS PARAGRAPH ALSO MUST BE REPORTED IMMEDIATELY TO ONE OF THE INDIVIDUALS LISTED ON THE SPONSOR CONTACT INFORMATION PAGE.*

**NOTE: Serious adverse experiences that do not reflect a potential study endpoint and that occur more than 14 days after discontinuation of the blinded study drug (i.e., etoricoxib or diclofenac sodium), and are not considered to be related to the blinded study drug, will not be reported to the SPONSOR or recorded on the worksheets as serious adverse experiences. However, events that may represent potential study endpoints and that occur any time up to the End of Study will be reported to the SPONSOR according to instructions provided in Section I.E.2.f**

*ALL PATIENTS WITH SERIOUS ADVERSE EXPERIENCES MUST BE FOLLOWED UP FOR OUTCOME.*

|  | | or | |  | | |
| --- | --- | --- | --- | --- | --- | --- |
|  | |  | |  | | |
|  | |  | |  | | |
|  | |  | |  | | |
|  | |  | |  | | |
|  | |  | |  | | |
| Telephone – Office: |  | |  | | Telephone – Office: |  |
| FAX No.: |  | |  | | FAX No.: |  |
| 24 Hour Contact: |  | |  | | 24 Hour Contact: |  |
| or | |  | |  | | |
|  | | or | |  | | |
|  | |  | |  | | |
|  | |  | |  | | |
|  | |  | |  | | |
|  | |  | |  | | |
|  | |  | |  | | |
| Telephone – Office: |  | |  | | Telephone – Office: |  |
| FAX No.: |  | |  | | FAX No.: |  |
| 24 Hour Contact: |  | |  | | 24 Hour Contact: |  |

^†^ See Protocol Section I.E2f., Safety Measurements, for definitions of serious adverse experiences.

#

## I. CLINICAL SECTIONS

A. CONTEXT AND RATIONALE

**1. Introduction**

The Rotator Cuff of the Shoulder consists of the supraspinatus, infraspinatus, teres minor and subscapular muscles. Normally, the rotator cuff slides under the coracoacromial arch through the subacromial deltoid bursa. Any interference with that mechanism results in a subacromial conflict.

A review of the literature on the subject shows that two pathophysiological theories coexist: the theory of subacromial conflict or 'impingement syndrome' by Neer (1,2) and the degenerative theory proposed by several authors (3-5).

Indeed, the factors which may interfere with the normal biomechanics of the rotator cuff are an irregular appearance of the acromial arch (osteoarthritis, sequela of fracture, hypertrophic morphology of the acromion) (6,7), loss of the slip properties of the leaflets of the subacromial bursa, a traumatic lesion of the cuff muscles, in particular the muscles contributing to lowering the head of the humerus, and excessive use of the shoulder. Intrinsic wear of the tendons over time may contribute to the emergence of subsequent lesions (8-10). Anoxic phenomena due to disorders of the local microvascularization may also contribute concomitantly.

At an early stage (Neer stage 1), cuff lesions are characterized by reversible edema and hyperemia (1). That stage consists in simple tendinitis of the shoulder. Stage 2 in Neer's classification reflects intra-tendinous degeneration with fibrosis. In stage 3, incomplete- or complete-thickness tears of the rotator cuff occur.

Diseases of the shoulder tendons not involving tear are extremely frequently encountered in everyday practice. The diagnosis is considered in the event of shoulder pain promoted by effort and partially regressing with warming up. A nighttime painful component is often observed. Physical examination does not evidence a limitation in active or passive movement of the shoulder. In the event of a subacromial conflict, abduction between 60 and 80° is painful. Non-steroidal anti-inflammatory drugs (NSAIDs) are the basis for symptomatic treatment of the disease (11).

**2. Value of selective COX-2 inhibitors**

Toxicity, particularly in the gastrointestinal (GI) tract, is a common occurrence with existing nonsteroidal anti-inflammatory drugs (NSAIDs). Overall, NSAID gastropathy (GI bleeding, ulcer, perforation) is considered the most common serious (and life threatening) adverse drug event among patients in industrialized nations. Furthermore, GI intolerance (e.g., dyspepsia) frequently necessitates discontinuation of NSAID therapy and triggers expensive evaluation and treatment. Thus, NSAID intolerability is a significant source of morbidity in the treatment of arthritis and other inflammatory disorders, resulting in the use of expensive health-care resources.

COX-2 selective inhibitors were developed with the hypothesis that they would provide the anti-inflammatory and analgesic efficacy of non-selective NSAIDs, but with a reduced incidence of gastrointestinal (GI) mucosal injury, based on their lack of inhibition of COX-1, which is involved in the physiologic processes that protect the gastric mucosa. (12) Clinical selectivity has been based on either surrogates for clinical toxicity (e.g., endoscopically visualized gastroduodenal ulcers) or actual clinical endpoints at anti-inflammatory doses of an inhibitor. In published literature, selective COX-2 inhibition has been shown to result in significantly less GI toxicity compared to nonselective NSAIDs including ibuprofen, diclofenac, and naproxen in various clinical models, thus confirming the hypothesis(13). During the VIGOR study, with mean follow-up of 9 months, 2.1 gastrointestinal events ( PUBs) were observed in 100 patient-years with rofecoxib compared to 4.5 per 100 patient-years with naproxen (relative risk 0.5; 95% confidence interval [0.3 - 0.6]; p < 0.001) (14).

The administration of rofecoxib, a selective inhibitor of COX-2, may have beneficial therapeutic effects in patients with Rotator Cuff Syndrome while significantly reducing the incidence of gastrointestinal toxicity related to conventional NSAIDs which are non-selective inhibitors of both COX-1 and COX-2.

The main objective of the study is thus to compare the short-term efficacy of rofecoxib at a dosage of 50 mg qd. in the treatment of acute painful Rotator Cuff Syndrome versus placebo and diclofenac 50 mg t.i.d.

**3. Rationale for Dose selection**

The selection of the rofecoxib dosage (Appendix 1) for this study was based on the efficacy data from the clinical trials and the experience already acquired with the drug in other diseases. Thus, the recommended dosages for the treatment of osteoarthritis and rheumatoid arthritis are 12.5 or 25 mg/qd and 25 mg/qd of rofecoxib, respectively.(15,16) The acute painful phenomena observed in the course of dysmenorrhea, post-operatively or during dental care, were alleviated by an optimal dosage of 50 mg/d of rofecoxib (17-18). The acute painful syndrome related to disease of the Rotator Cuff is closer to those painful mechanisms providing the rationale for a dosage of 50 mg/qd of rofecoxib in this study.

Diclofenac, at a dosage of 50 mg t.i.d., is frequently used worldwide for the treatment of abarticular diseases. The administration of 3 divided doses daily is compatible with the known pharmacokinetic profile of the NSAID and in line with the marketing authorization recommendations (Appendix 2).

**B. OBJECTIVES**

**1. Primary objective**

The primary objective is to compare the short-term symptomatic efficacy of rofecoxib at a dosage of 50 mg q.d. (pain measured on a Numerical Rating Scale - NRS - from 0 to 10) versus placebo. Efficacy is defined the mean changes in pain (night pain + pain during physical activities/2) from D0 to D7.

**2. Secondary objectives**

- Changes in pain during the physical activities (NRS)
- Changes in pain at night (NRS)
- Changes in functional index at D0 and D7
- Changes in patient’s global assessment of disease activity
- Patient’s global assessment of the response to the treatment at D7
- Patient’s global assessment of the level of state at D7
- Requirement of a local injection of corticosteroid in the shoulder during the 7 days of the study including the last visit at D7
- Number of acetaminophen/paracetamol tablets per day
- Tolerability

The second secondary objective is to evaluate the safety of rofecoxib at a dosage of 50 mg q.d. versus diclofenac 50 mg t.i.d. in the treatment of acute painful Rotator Cuff Syndrome.

C. HYPOTHESES

Primary hypothesis

Rofecoxib 50 mg q.d will demonstrate superior efficacy than placebo in the treatment of the acute painful phase of Rotator Cuff Syndrome.

**D. PATIENT POPULATION DEFINITION**

**1. Inclusion criteria**

- Male or female
- aged less than 60 years
- with a clinical diagnosis of Rotator Cuff Syndrome: painful Jobe’s test (see appendix 3)
- onset of the current episode less than 7 days prior the first visit
- acute painful flare is required (NRS > 5).
- In the investigator's judgement, patient requires NSAID or COX-2 selective inhibitor therapy for at least 7 days.

It is to be noticed that cases of chronically painful shoulder with an abrupt resurgence of pain as per the criteria indicated above are suitable for inclusion in the protocol.

- Patient understanding the study procedures and agreeing to participate in the study by giving written informed consent.

**2. Exclusion criteria**

- Diagnosis of Rotator Cuff Tear (positive Jobe's maneuver - Appendix 3) and therefore a case not fulfilling the inclusion criteria.
- Patient presenting with limitation of external rotation in the physical examination compatible with advanced osteoarthritis or retractile capsulitis.
- Patient having taken NSAIDs in the 72 hours preceding the study and/or acetaminophen/paracetamol in the 12 hours preceding the study and/or having received a local corticosteroid injection in the 90 days preceding the study.
- Patient presenting with a concomitant medical or rheumatological disease liable to influence or interfere with the assessment of efficacy, including, in a non-restrictive manner, inflammatory rheumatism (e.g. rheumatoid arthritis, systemic lupus erythematosus, vertebral joint disease, polymyalgia rheumatica).
- Patient presenting with active peptic ulcer or gastrointestinal bleeding.
- Female patient if pregnant or nursing.
- Patient with a history of inflammatory bowel disease .
- Patient known as having impaired renal function, defined as estimated creatinine clearance less than 30 mL/min.
- Patient having severe congestive heart failure.
- Patient known as having moderate to severe liver failure (Child-Pugh score > 9).
- Allergic patient or patient having hypersensitivity (asthma, acute rhinitis, nasal polyps, angioedema or urticaria) to aspirin, diclofenac, other NSAIDs or COX-2 selective inhibitors.
- Patient unable to understand and complete the study questionnaire.

**E. STUDY DESIGN**

**1. Summary of study design**

This is a randomized, double-blind, parallel-group study to evaluate the efficacy and safety of rofecoxib (50 mg q.d.) vs. placebo and diclofenac (50 mg t.i.d.) in the treatment of patients with acute painful Rotator Cuff Syndrome. The Rotator Cuff Syndrome will be diagnosed using standard clinical criteria. The baseline data will be collected during screening/randomization visit. The efficacy and safety data will be collected after 7 days of treatment.

The assessment of the safety of rofecoxib at a dosage of 50 mg q.d and diclofenac 50mg t.i.d over 7 days will be conducted by determining the incidence of treatment discontinuations due to clinical gastrointestinal adverse events or spontaneously reported adverse events.

1. **Treatments**
2. **Treatment regimen**

Eligible patients will be randomized to one of the three treatment groups and receive either rofecoxib 50 mg q.d. (N = 82) or either diclofenac of 50 mg t.i.d.
(N = 82) or placebo (N = 82) by the oral route for 7 days. ***Supplies will be provide for a treatment period of 9 days in case of a patient who cannot return to the last visit exactly after 7 days.*** All the drugs will be taken daily according to the following treatment regimen:

| **Treatment** | **Treatment composition** |
| --- | --- |
| Rofecoxib, 50 mg q.d. | 1 rofecoxib tablet q.d. in the morning  1 diclofenac 50-mg placebo tablet t.i.d. |
| Diclofenac, 50 mg t.i.d. | 1 diclofenac 50-mg tablet t.i.d.  1 rofecoxib placebo tablet q.d. in the morning |
| Placebo | 1 rofecoxib placebo tablet q.d. in the morning  1 diclofenac 50-mg placebo tablet t.i.d. |

The patients will be asked to take the tablets at the same times each day, in the morning, at noon and at night.

The first study medication intake will take place in the rheumatologist’s office in the presence of the physician (cf. Procedures).

**b. Prior and concomitant medication(s)/treatment(s)**

NSAIDs are prohibited with the exception of use of aspirine of low dose (at platelet-aggregation inhibiting dosages) for cardioprotective benefit. All previous NSAID treatments are to have been discontinued at least 72 hours prior to inclusion of the patient in the study.

Use of H_2_ blockers, antiacids and or proton-pump inhibitors is permitted during the study.

Patients may pursue or initiate a treatment other than the study medication during the study.

The standard precautions will be observed for treatments liable to interfere with rofecoxib
(cf. Appendix 1).

Patients will be permitted to use acetaminophen/paracetamol on an “as needed” as rescue therapy to alleviate unbearable shoulder pain at any time during the study. The maximum daily dose of acetaminophen/paracetamol is restricted to 4000 mg/d.

All previous and concomitant treatments will be reported in the case report forms during each visit.

**c. Treatment compliance**

Satisfactory compliance with treatment during the study is required of the patients. Compliance will be checked by counting the tablets during visit 2.

1. **Data collected during the study**
2. **Demographic data**

The following data will be collected: age, gender, weight, height, dominant side (right- or left-handed) at visit 1

1. **Disease characteristics**

The following information will be reported at visit 1

1. date of symptom onset
2. painful side (right or left)
3. painful condition before the acute episode (continuous presence, present but intermittent, absent). In the event of intermittent painful phases, the date of the last painful acute flare will be reported. The information will concern the shoulder for which the patient is consulting at the time of study inclusion.
4. **Medical history**

The concomitant diseases for which the patient is monitored will be reported. A list of the diseases liable to constitute an exclusion criterion for the study will be validated in the case report form (cf. section I.D.2)

1. **Concomitant medications**

An exhaustive list of the medications taken by the patient will be compiled. In addition, a list of the medications liable to constitute a study exclusion criteria will be validated in the case report form (cf. section E.2.b).

1. **Efficacy criteria**

###### Pain assessment

Pain during daily activities will be validated on a 10 NRS on which 0 represents the absence of pain and 10 the worst pain imaginable.

**Functional impairment assessment** (Neer’s functional index)

During visits 1 (screening/inclusion) and 2 (after 7 days of treatment), the patient will be asked to assess some daily activities using the target upper limb. The investigator will report the responses to an interview consisting in 10 questions relating to the activities of everyday life.

The score 0 will be assigned if the function can be achieved normally without difficulty, 1 with some difficulty, 2 with marked difficulty, 3 with great difficulty (requiring assistance) and 4 in the event of inability.

The items in the ‘functional’ domain of Neer’s index are as follows :

1. Put a hand in the back-pocket of a garment
2. Intimate hygiene
3. Wash the opposite arm pit
4. Eat with a knife and fork
5. Comb hair
6. Use the hand with the arm at shoulder height
7. Carry 500 to 750 g with the arm against the body
8. Get dressed
9. Sleep on the side
10. Accomplish usual work (if impossible, specify the conditions)

**Global assessment of disease activity by the patient**

At visits 1 (screening/inclusion) and 2 (after 7 days of treatment), the patient will be asked to assess disease activity using the following scale:

“considering all the ways your disease of your shoulder affects you, mark an (X) in a boxfor how well you are doing”:

0 = very well

1 = well

2 = fair

3 = Poor

4 = Very poor

**Assessment of night pain by NRS**

Night pain will be considered to reflect the inflammatory process. Such information will be reported using a Numerical Rating Scale (NRS) on which 0 represents the absence of night pain and 10 the worst night pain imaginable.

###### Patient’s global assessment on her/his relative condition

At the end of the study **Patient’s global assessment on her/his relative condition** will be compared to baseline using a 15 grade scale by asking the patient the following question: ‘How do you assess your relative condition when compared to prior therapy”

-7 = very great deal worse

-6 = great deal worse

-5 = good deal worse

-4 = moderately worse

-3 = somewhat worse

-2= little worse

-1 = hardly worse

0 = unchanged

+1 = hardly better

+2 = little better

+3 = somewhat better

+4 = moderately better

+5 = good deal better

+6 = great deal better

+7 = very great deal better

Patient’s global assessment of his/her current condition

A binary variable (yes/no) will be used in response to the following question: “Taking into account all your daily activities and considering not only the pain but also the functional disability, do you consider that your current condition is satisfactory ?”

Modalities of patient response to the questionnaires

The investigators are not to assist patients in answering the questionnaire. Patients are to respond based on the instruction answer on the basis of the instructions provided on the appropriate section of the case report form. Patients are to complete the questionnaire without the assistance of friends or family. The study investigators are responsible for ensuring that the questionnaire is completed during the required visits. The patients must never be able to refer to the answers given in previous questionnaires.

###### Numbers of acetaminophen/paracetamol tablets per days

**Requirement of a local corticosteroïd injection in the shoulder during the 7 days of the study, including the last visit at D7.**

1. Safety measurements

**Collection of adverse events**

The adverse events considered clinically pertinent by the investigator will be reported in the case report forms.

An adverse event is defined as any unfavorable and unintended change in the structure, function, or chemistry of the body temporally associated with the use of the SPONSOR’S product, whether or not considered related to the use of the product. Any worsening (i.e., any clinically significant adverse change in frequency and/or intensity) of a preexisting condition which is temporally associated with the use of the SPONSOR’S product, is also an adverse experience.

**Serious adverse events**

A serious adverse experience is any adverse event occurring at any dose that :

- †Results in death; or
- †Is life threatening
- †Results in a persistent or significant disability/incapacity (substantial disruption of one’s ability to conduct normal life functions); or
- †Results in or prolongs an existing inpatient hospitalization
- †Is a congenital anomaly/birth defect (in offspring of taking the product regardless of time to diagnosis); or
- Is a cancer; or
- Is the result of an overdose (whether accidental or intentional).

**ALSO:**

Other important medical events that may not result in death, not be life threatening, or not require hospitalization may be considered a serious adverse experience when, based upon appropriate medical judgment, the event may jeopardize the And may require medical or surgical intervention to prevent one of the outcomes listed previously (designated above by a †).

###### Reporting

Adverse events will be reported by the investigator.

All the adverse events occurring during the week of follow-up in the set of patients included are to be reported by the investigator, irrespective of whether or not the adverse event appears related to the treatment.

If the event consists in a non-serious adverse event, the investigator will report it in the case report form with all the information on the subject that he is able to obtain.

If the event consists in a serious adverse event, the investigator is required to report it within 24 hours of becoming aware of it. The initial contact is the person responsible for the pharmacovigilance of the study:

1. CRO
2. Doctor Marie-Blanche DUCROCQ

##### Tel.: 01 47 54 88 96 – Fax: 01 47 54 85 27

Address: MERCK SHARP & DOHME-CHIBRET

3, Avenue Hoche

75114 PARIS Cedex 08

### F. STUDY PROCEDURES

**1.Visit 1 – Screening/Inclusion**

| **List of procedures** |
| --- |
| Review Entry Criteria  Informed consent  Medical history  Prior and concomitant medications  Physical examination (with body weight, height and blood pressure determination)  Assessment of pain during daily activities pre-treatment  Assessment of night pain using the NRS  Assessment of the functional impairment (Neer’s functional index)  Patient’s global assessment of disease activity  Provide patient’s diary  Dispense Study medication  Schedule Visit 2 |

The patients eligible for inclusion in the study will be evaluated in order to determine whether they meet the inclusion criteria listed in section I.D. The criteria include confirmation of the diagnosis of Rotator Cuff Syndrome in an acute painful phase requiring NSAID or a COX-2 selective inhibitor therapy for at least 7 days. Investigation for the signs of Rotator Cuff Tear (Appendix 3) or Retractile Capsulitis is to be conducted and those signs are required not to be present in order to include the patients in the study.

Written informed consent will be obtained in writing prior to the performance of any study- procedure

The patient’s medical history will be reported.

The patient will be questioned on the medications taken in the 90 days preceding visit 1.

A physical examination will be conducted and will include body weight, height and blood pressure determination.

Assessment of pain pre-treatment will be conducted by NRS. The self-assessment diary will be given to the patient and the modalities of pain assessment will be explained:

- the pain NRS is to be scored in the morning and evening at set times over the 7 days of the study
- the morning NRS will be used to report the intensity of the pain experienced during the previous night
- the evening NRS will report the pain experienced during the physical activities of that day.

The first two pain intensity scores will be assessed in the rheumatologist’s office in the latter’s presence and reported in the patient’s self-assessment diary:

- the first determination will report pain intensity during the daily activities in the day preceding the visit (NRS)
- the second will report the intensity of the pain experienced during the night preceding the visit (NRS).

Functional disability will be assessed using Neer’s functional index and a 5-grade Likert scale.

The patient’s global assessment of disease activity will be reported.

Study medication dispensing:

Each rheumatologist taking part in the study will have previously received blocks of 6 treatments. Each new patient included will receive the treatment bearing the lowest number. The number will be reported in the case report form. The patient will be given a pack containing two bottles of study medication (bottle A and B) and analgesics consisting in 500-mg acetaminophen/paracetamol tablets (tablets for a duration of 7 days).

The patients will take 1 tablet from bottle A and 1 tablet from bottle B, with 200 ml of water, in the rheumatologist’s presence on the day of inclusion.

If the visit takes place before 1 P.M., the second and third intakes will take place 6 and 12 h after the first intake on the same day. If the visit takes place after 1 P.M., a single intake will be taken on the evening of the same day and the next intake will be taken the next morning.

The patient will be free to use or not use the acetaminophen/paracetamol over the 7 days of the study. The number of tablets taken and the intake times will be reported in the patient’s self-assessment diary.

An appointment will be made for visit 2 (after 7 days of treatment), which will take place 7 days later.

**2.Visit 2 – After 7 days of treatment**

| **List of procedures** |
| --- |
| Collect the patient’s diary  Collect information on the adverse events and concomitant medications  Physical examination (including body weight, height and blood pressure determination)  Assessment of pain during daily activities during the day if not previously assess by the patient in her/his diary the day of the visit  Assessment of night pain using the NRS if not previously assess by the patient in her/his diary for the night preceding the visit  Assessment of functional impairment(Neer’s functional index)  Patient’s global assessment of disease activity (PGADS)  Patient’s global assessment of relative condition  Patient’s global assessment of current condition  Collect study medications / Perform tablet count |

Bottles A, B and acetaminophen/paracetamol will be collected. The tablets will be counted and the results reported, including acetaminophen/paracetamol.

Patients may resume taking NSAID, a COX-2 selective inhibitor or acetaminophen/paracetamol as of the day after visit 2 if the physician considers it appropriate.

Patients who do not attend visit 2 will be contacted. The study withdrawal procedure will then be applied.

**3. Study-discontinuation visit**

For randomized patients unable to complete the study until the end of the treatment phase of duration 7 days, for any reason, a study discontinuation visit is required.

The visit will be scheduled within 48 h of patient discontinuing from the study. The primary reason for discontinuation will be noted (e.g.: lack of efficacy, adverse event, etc.).

The patients may withdraw from the study at any time or the investigator may decide to withdraw a patient from the study if an adverse event occurs.

Procedures implemented during the study discontinuation visit

Question the patient on the adverse events and concomitant medications used since visit 1.

Conduct a physical examination and collect all the outcomes variables such as visit 2

The primary reason for discontinuation from the study, mainly lack of efficacy, serious adverse event, non-compliance or other (with an explanation) will be reported by the investigator.

Bottle A, B and acetaminophen/parcetamol will be collected. The tablets will be counted and the numbers reported.

**4. Unblinding procedure**

The study is a double-blind design (sponsor's in-house blind conditions). Neither the patient nor the physician nor the sponsor will be aware to which treatment group to which the patient has been assigned. The well-being of the patients taking part in the study will always take priority over any other consideration when the decision as to whether or not to break the blind is to be taken.

The investigator will not break the blind for a patient without the study monitor's approval.

**5.Non-compliance with treatment**

Patients who miss more than 20% of intakes will be considered noncompliant with treatment.

G. DATA ANALYSIS

**1. Responsibility for the analyses/In-house blind**

The statistical analysis of the data generated in the study will be conducted under the responsibility of Professor Philippe RAVAUD for the efficacy analyses and of MSD-Chibret Laboratory for the safety data. For the purpose of the final analysis, the official database will not be unblinded until medical/scientific review has been completed, protocol violators have been identified and the data have been declared 'clean'.

**2. Variables analyzed**

- 1. **Primary variable**

The main analysis will evaluate the mean changes in pain (night pain + pain during physical activities/2) from D0 to D7. The first value will be recorded by the physician at baseline during the first visit. The following values will be recorded in the evening for pain NRS during the daily activities and the next morning for night pain. As a result, D0 will assess the pain recorded before treatment and D1 will assess the mean level of pain recorded the day prior to the study + the morning on the 1^st^ day of the study. The main effectiveness analysis will be a linear mixed model for repeated measures

- 1. **Secondary analyses**

Descriptive analyses of linearity over the course of time of pain during daily activities, linearity over the course of time of night pain and linearity of the number of paracetamol tablets taken during the study will be done for each treatment group.

Variations over the course of time in pain during daily activities and in night pain will be studied, in order to determine which treatment group (rofecoxib or placebo) enabled greater reduction of pain.

Similarly, variations in the number of paracetamol tablets taken over the course of time will be studied, in order to determine which treatment (rofecoxib or placebo) led to less need for paracetamol.

Use of a local steroid injection in the shoulder evaluated in the rofecoxib group during the study or at the end of the final visit will be compared with that in the placebo group (Chi2 test). It will be described for the 3 treatment groups.

Absolute variation in Neer index between D0 and D7 in the rofecoxib group will be compared with that in the placebo group (Student’s test). This variation will be described for the 3 treatment groups.

Percentages of patients in the rofecoxib group will be compared with those in the Placebo group having answered the various modalities of the following criteria (Chi2 test):

- Global assessment by patient of disease activity;
- Global assessment by patient of current status;
- Global relative assessment by patient of response to treatment.

Numbers and percentages of patients having answered the various modalities of these criteria will be described for the 3 treatment groups.

**3. Risks of error**

A power of 80% is required for the primary analysis. The first-order error has been set at 5%.

**4. Hypothesis**

Rofecoxib 50 mg q.d., will demonstrate superior efficacy than placebo in the treatment of Rotator Cuff Syndrome, in the acute painful phase, over a follow-up period of 7 days.

On the basis of the published data which present the results as the mean change in NRS pain score for a group of patients, the mean change in VAS pain score is 30 mm and the mean end-of-study VAS pain score is 40 mm with a standard deviation of 20 mm in the group receiving an NSAID (19). The percentage success rate for the treatment group will therefore be of the order of 40% and only 20% in the placebo group.

**5. Sample size calculation**

In order to evidence a 20% mean difference between the treatment and placebo groups
(40% response in the treatment group vs. 20% in the placebo group), with a first-order error of 5% and a power of 80%, 82 subjects will be required per group, i.e. 164 patients for the two treatment groups. In order to obtain uniform numbers of patients in the treatment groups, an equivalent number of patients will be included in the diclofenac group, i.e. a total of 240 patients presenting with rotator cuff syndrome (82 patients per treatment arm).

**6. Management of missing data**

**For the primary variable:**

Any patient discontinued from the study for inefficacy will be considered a failure: in statistical terms, for the survival curve, the patient will be maintained in the analysis for the 7 days of the study but not be considered as an event or a lost of follow-up.

Any patient discontinued from the study for a reason other than inefficacy will be considered lost to follow-up at the time of discontinuation from the study for the survival curve.

If one of the variables (NRS) is missing (for example. night NRS for the morning of day 4), the previous night NRS value will be taken.

If several variables are missing through to the end of the study, the last observation carried forward (LOCF) method will be conducted and consist in replacing the missing data by the last known value, including that on D0 if only that value has been reported.

**For the secondary variables:**

The same method will be applied to the secondary variables studied independently (night pain NRS, pain during daily activities NRS) when one or more data items are missing. The previous value or the last known value will be carried forward.

**II. administratiVE AND REGULATORY sections**

**A. LABELING, PACKAGING, STORAGE AND RETURN OF STUDY MEDICATIONS**

**1. Product Descriptions**

The SPONSOR will supply the study medications listed in the table below. Acetaminophen will be used as rescue medication but also as a secondary efficacy criterion and will be supplied by the sponsor.

| **Product** | **Potency** | **Dosage form** | **Manufacturer** | **Storage conditions** |
| --- | --- | --- | --- | --- |
| **Rofecoxib** | **50 mg** | **Tablet** |  |  |
| **Rofecoxib 50 mg placebo** | **Placebo** | **Tablet** |  |  |
| **Diclofenac** | **50 mg** | **Tablet** |  |  |
| **Diclofenac 50 mg placebo** | **Placebo** | **Tablet** |  |  |
| **Acetaminophen/Paracetamol** | **500 mg** | **Tablet** |  |  |

**2. Primary Packaging and labeling Information**

The following medications will be supplied as indicated below:

| **Bottle** | **Product name and potency** | **Fill count** | **Dosing instructions** |
| --- | --- | --- | --- |
| **A** | **Rofecoxib 50 mg or placebo** | **9 tablets** | **Take 1 tablet in the morning** |
| **B** | **Diclofenac 50 mg or placebo** | **27 tablets** | **Take 1 tablet each morning, noon and night** |
|  | **Acetaminophen/paracetamol 500 mg** | **72 tablets** | **As required without exceeding 4000 mg/day (8 tablets per day)** |

**For the investigators (rheumatologists)**

The treatments will be supplied in blocks of 6 packs each containing bottle A, B and acetaminophen/paracetamol. Each bottle will bear a standard label. The information reported on the label will be presented as indicated below. The investigator will report the patient's randomization number.

| - **Space for the randomization number** - **Component ID** - **Number of tablets** - **Packaging control number** - **Compound identification - Protocol number (if required)** - **Instructions for use** - **Storage conditions** - **Regulatory requirements** - **SPONSOR's address** |
| --- |

**3. Secondary Packaging and Labeling Information**

The medications will be supplied in treatment packs. During the inclusion visit
(visit 1) when the medication is dispensed, each patient will receive one pack containing Bottle A, Bottle B and acetaminophen/paracetamol. The treatments are shown by treatment group in the table below.

| **Group** | **BottleA** | **Bottle B** |  |
| --- | --- | --- | --- |
| **rofecoxib 50 mg**  **q.d.** | **9 rofecoxib 50 mg**  **tablets** | **27 diclofenac 50 mg placebo tablets** | **72 Acetaminophen/paracetamol 500 mg tablets** |
| **diclofenac 50 mg**  **t.i.d.** | **9 rofecoxib 50 mg placebo tablets** | **27 diclofenac 50 mg tablets** | **72 Acetaminophen/Paracetamol 500 mg tablets** |
| **Placebo** | **9 rofecoxib 50 mg placebo tablets** | **27 diclofenac 50 mg placebo tablets** | **72 Acetaminophen/paracetamol 500 mg tablets** |

Each treatment pack will bear a standardized label. The labeling information will be presented as follows. The investigator will indicate the patient's randomization number.

| - **Space for the randomization number** - **Component ID** - **Bottle identification** - **Pack contents** - **Packaging control number** - **Compound identification - Protocol number** - **Storage conditions** - **SPONSOR's address** |
| --- |

**4. Standard procedures / Drug accounting / Comparator statement**

Investigational clinical supplies must be received by a designated person at the study site, handled and stored safely and properly, and kept in a secured location to which only the investigator and designated assistants have access. Clinical supplies are to be dispensed only in accordance with the protocol. The investigator is responsible for keeping accurate records of the clinical supplies received from the SPONSOR, the amount dispensed to and returned by the subjects/patients, and the amount remaining at the conclusion of the study. In accordance with Good Pharmacy Practices, gloves should always be worn by study personnel if directly handling tablets or capsules that are returned (i.e., when counting returns). The Clinical Monitor should be contacted with any questions concerning investigational products where special or protective handling is indicated. At the end of the study, all clinical supplies including partial and empty containers must be returned as indicated on the Contact Information page(s).

The centers will contact Merck's local personnel to obtain the appropriate documentation to be completed for drug accounting.

The investigator or his appointed assistant will not open the individual bottles of drugs to count the tablets/capsules, etc., prior to dispensing them to patients. Any deviation from that provision is to be discussed with the study monitor.

## B. CLINICAL DATA COLLECTION

### Method of Data Collection

Workbooklets will be provided by the SPONSOR to record data in the clinic. Data on workbooklets may be handwritten.

Periodically, a representative of the SPONSOR will review study documents (see Protocol Section Study Documentation and Records Retention) to verify compliance with the protocol. The SPONSOR representative will also review the accuracy of the data compared to source documents.

After preliminary review of these worksheets by the Investigator/study staff and by the SPONSOR representative, copies of the worksheets will be sent to the SPONSOR or SPONSOR representative for entry into a database. Original worksheets will remain at the site as source/support documents.

As a result of the SPONSOR or SPONSOR representative, data review process, corrections or changes to the data may be required. Discrepancies or questions concerning the data will be sent to the Investigator. The discrepancy reports should be resolved by the Investigator/study staff; signed and dated, and a copy returned to the SPONSOR or SPONSOR representative. The original discrepancy report must be retained in the subject/patient binder as a record of changes or acknowledgment of the receipt of queries on the data.

**C. STUDY DOCUMENTATION AND RECORDS RETENTION**

Study documentation includes all case report forms, data correction forms, workbooks, source documents, monitoring logs and appointment schedules, SPONSOR-investigator correspondence and regulatory documents (e.g., signed protocol and amendments, Independent Ethics or Institutional Review Committee correspondence and approval, approved and signed consent forms, Statement of Investigator form, clinical supplies receipts, and distribution records).

Source documents include all recordings of observations or notations of clinical activities and all reports and records necessary for the evaluation and reconstruction of the clinical research study.

Patient-generated source documents (e.g., questionnaires, etc.) are to be generated only by the patient, reviewed by the study coordinator/investigator upon completion, and initialed and dated by the patient to confirm the accuracy of the recorded information.

Government agency regulations and directives require that all study documentation pertaining to the conduct of a clinical trial must be retained by the investigator. They shall be retained until at least 2 years after the last approval of a marketing application in an International Conference on Harmonisation (ICH) region. The SPONSOR will notify the investigator in writing when retention is no longer necessary.

## INFORMED CONSENT

In compliance with the Declaration of Helsinki and the French law dated December 20, 1988, the investigator must obtain documented consent from each potential in biomedical research or when an investigational drug is administered to in a clinical study.

Consent must be documented by the dated signature on a Consent Form along with the dated signature of the person conducting the consent discussion.

A copy of the signed and dated consent form (along with the PPI if appropriate) should be given to the before participation in the trial.

The initial informed consent form and any subsequent revised written informed consent form, and written information must receive the IRB/IEC’s approval/favorable opinion in advance of use. The or his/her legally acceptable representative should be informed in a timely manner if new information becomes available that may be relevant to the willingness to continue participation in the trial. The communication of this information should be documented.

The investigator will certify having obtained the patient's freely given informed consent in the case report form.

## INSTITUTIONAL REVIEW BOARD (IRB)/INDEPENDENT ETHICS COMMITTEE (IEC)

The primary investigator is responsible for obtaining Review Board approval of the protocol, as well as approval of all subsequent major changes, in compliance with local

law. Copies of these approvals must be forwarded to the SPONSOR. Particular attention is drawn to the International Conference on Harmonization (ICH) Guidelines for Good Clinical Practices for Institutional Review Board/Independent Ethics Committees, and a copy of the guidelines is attached to this protocol.

## CONFIDENTIALITY

By signing this protocol, the investigator affirms to the SPONSOR that information furnished to the investigator by the SPONSOR will be maintained in confidence and such information will be divulged to the Institutional Review Board, Ethical Review Committee, or similar or expert committee; affiliated institution; and employees only under an appropriate understanding of confidentiality with such board or committee, affiliated institution and employees. Data generated by this study will be considered confidential by the investigator, except to the extent that it is included in a publication as provided in Section Publications

### Confidentiality of Records

By signing this protocol, the investigator agrees that the SPONSOR (or SPONSOR representative), Institutional Review Board/Independent Ethics Committee (IRB/IEC) or Regulatory Agency representatives may consult and/or copy study documents (see Protocol Section  Study Documentation and Records Retention) in order to verify case report form data. By signing the consent form, the agrees to this process. If study documents will be photocopied during the process of verifying case report form information, the will be identified by unique code only; full names/initials will be masked.

### Confidentiality of Investigator Information

By signing this protocol, the investigator recognizes that certain personal identifying information (e.g., name, hospital or clinic address, curriculum vitae) may be made part of a regulatory submission and may be transmitted (either in hard copy or electronically) to all Merck subsidiaries/agents worldwide for internal study management purposes or as required by individual regulatory agencies. Additionally, the investigator’s name, hospital/clinic address/phone number may be included when reporting certain serious adverse events to regulatory agencies or to other investigators.

## COMPLIANCE WITH LAW, AUDIT, AND DEBARMENT

By signing this protocol, the investigator agrees to conduct the study in an efficient and diligent manner and in conformance with this protocol; generally accepted standards of Good Clinical Practice; and all applicable federal, state, and local laws, rules and regulations relating to the conduct of the clinical study.

The investigator also agrees to allow monitoring, audits, Institutional Review Board/Independent Ethics Committee review and regulatory agency inspection of trial-related documents and procedures and provide for direct access to all study-related source data and documents.

The investigator shall prepare and maintain complete and accurate study documentation in compliance with Good Clinical Practice standards and applicable federal, state, and local laws, rules and regulations; and, for each participating in the study, provide all data, and upon completion or termination of the clinical study submit any other reports to the SPONSOR as required by this protocol or as otherwise required pursuant to any agreement with the SPONSOR.

Study documentation (see Protocol Section IIC, Study Documentation and Records Retention) will be promptly and fully disclosed to the SPONSOR by the investigator upon request and also shall be made available at the investigator’s site upon request for inspection, copying, review and audit at reasonable times by representatives of the SPONSOR or any regulatory agencies. The investigator agrees to promptly take any reasonable steps that are requested by the SPONSOR as a result of an audit to cure deficiencies in the study documentation and case report forms.

In the event the SPONSOR prematurely terminates a particular trial site, the SPONSOR will promptly notify that site’s IRB/IEC.

## QUALITY CONTROL AND QUALITY ASSURANCE

By signing this protocol, the SPONSOR agrees to be responsible for implementing and maintaining quality control and quality assurance systems with written SOPs to ensure that trials are conducted and data are generated, documented, and reported in compliance with the protocol, accepted standards of Good Clinical Practice, and all applicable federal, state, and local laws, rules and regulations relating to the conduct of the clinical study.

###### I. PARTIE’S OBLIGATIONS

**a. Obligations incumbent on MSD-Chibret**

MSD-Chibret will assume the obligations conferred by the French law dated December 20,

1988, known as the “Huriet Law”, and, in particular:

▪ the obligation of taking out insurance cover for the sponsor's third party liability and that of all contributors (Appendix 6)

▪ the obligation of forwarding a letter of intent to the AFSSAPS

▪ the obligation of compiling the patient information document provided for by law

▪ the obligation of informing AFSSAPS (Clinical Trials Unit) in the event of deaths or serious adverse events related to the medication.

In addition, pursuant to article L.4113-6 of the French Code of Public Health, , MSD-Chibret is required to forward the present protocol and all the financial conventions to the National Council of the Order of Physicians for an opinion.

**b.** **Obligations incumbent on the investigator**

The investigator undertakes to conduct the study in compliance with Good Clinical Practice and current regulations, particularly the provisions of the act dated December 20, 1988, relating to the protection of persons, and the decree dated March 13, 1995, relating to the organization of pharmacovigilance.

In compliance with those provisions, the investigator assumes, in particular, the following obligations:

▪ the obligation of checking that all the required conditions (assessment of the nature of the study, sufficient prior investigation, etc.) have been fulfilled in order to proceed with the study. The investigator may request additional information if he considers himself insufficiently informed;

- the obligation of satisfactorily completing the study entrusted to him;
- the obligation of informing the patients and obtaining their consent;

▪ the obligation of complying with Good Clinical Practice and the scientific protocol;

▪ the obligation of reporting any serious adverse event of which he becomes aware to the SPONSOR or SPONSOR representative within 24 hours;

▪ pursuant to article L 4113-9 and L 4113-10 of the French Code of Public Health, the obligation of declaring the present protocol and all the financial conventions to the Departmental Council of the Order of Physicians to which he reports.

The investigator will freely conduct the study provided for by the present protocol under his scientific responsibility.

For the conduct of the study, the investigator will be responsible for deciding the human and material resources to be committed to study implementation in compliance with statutory provisions.

**J. ADMINISTRATIVE ORGANIZATION OF THE STUDY**

The study will be conducted under the responsibility of the national coordinator, Professor Maxime DOUGADOS.

Centralized coordination of the study will be conducted by Doctor Isabelle LOGEART at MSD-Chibret's headquarters.

## PUBLICATIONS

As this study is part of a multicenter trial, publications derived from this study should include input from the principal investigator, his/her colleagues, the other investigators in this trial, and SPONSOR personnel. Such input should be reflected in publication authorship, and whenever possible, preliminary agreement regarding the strategy for order of authors' names should be established before conducting the study. Subsequent to the multicenter publication, or 24 months after completion of the study, whichever comes first, an investigator and/or his/her colleagues may publish the results for their study site independently.

The SPONSOR must have the opportunity to review all proposed abstracts, manuscripts, or presentations regarding this study 60 days prior to submission for publication/presentation. Any information identified by the SPONSOR as confidential must be deleted prior to submission, it being understood that the results of this study are not to be considered confidential. SPONSOR review can be expedited to meet publication guidelines.

# L. SIGNATURES

## SPONSOR’S REPRESENTATIVE

| TYPED NAME |  | SIGNATURE |  | DATE |
| --- | --- | --- | --- | --- |
| Dr Isabelle Logeart , Medical Manager |  |  |  |  |

## INVESTIGATOR

I agree to conduct this clinical study in accordance with the design and specific provisions of this protocol; deviations from the protocol are acceptable only with a mutually agreed upon protocol amendment. I agree to conduct the study in accordance with generally accepted standards of Good Clinical Practice. I also agree to report all information or data in accordance with the protocol and, in particular, I agree to report any serious adverse experiences as defined in Section I.G. of this protocol. I also agree to handle all clinical supplies provided by the SPONSOR and collect and handle all clinical specimens in accordance with the protocol.

| TYPED NAME |  | SIGNATURE |  | DATE |
| --- | --- | --- | --- | --- |
|  |  |  |  |  |
|  |  |  |  |  |

**LIST OF REFERENCES**

1. Neer C.S. Impingement lesions. Clin. Orthop. 1983, 173, 70-77.
2. Neer C.S., Craig E.V., Fukuda H. Cuff-tear arthropathy. J. Bone Joint Surg. 1983 ; 65A :1232-44.
3. Rathbun J.B., Macnab I The micovascular patterns of the rotator cuff. J. Bone and Joint Surg. 1970, 52-B, 540.
4. Uhthoff H.K., Lohr J., FarkAr K. The pathogenesis of rotator cuff tears. *In* : The shoulder. 211-212 Ed. N. Takagishi. Professional post-graduate services. Tokyo.
5. Yamanaka , Fukuda H. Pathological studies of the suprasupinatus tendon with reference to incomplete thickness tear. *In* The shoulder Ed N. Tagagishi. Professional post-graduate services. Tokyo.
6. Bigliani L.U., Morrison D.S., April E.W. Morphology of the acromion and its relationship to rotator cuff tears. Orthop. Trans. 1986 ;10 :459-60.
7. Ozaki J, Fujimoto S., Nakagawa Y., Masuhara K., Tamai S. Tears of the rotator cuff of the shoulder associated with pathological changes in the acromion. A study in cadavers. J. Bone Joint Surg. 1988 ; 70A : 1224-30.
8. Migrom C., Schaffler M., Gilbert S., Holsbeeck M . Rotator-cuff changes in asymptomatic adults. The effect of age, hand dominance and gender.
9. Palma A.F. Surgical anatomy of the rotator cuff and the natural history of degenerative periarthritis. Surg. Clin. North Am. 1967 ;43 :1507-20.
10. Sèze S. de, Ryckewaert A., Welfling J., Hubault A., Renier J.C., Caroit M. *et al*. Etudes sur l’epaule douloureuse. Rev. Rhum. 1961 ; 3 :85-94.
11. Klippel J.H., Dieppe P.A. Rotator cuff disorders. *In* : Textbook of rheumatology. Ed. Mosby. 1998.
12. Vane JR, Botting RM. Mechanism of action of anti-inflammatory drugs. Scand J Gastroenterol 1996;25(Suppl 102):9-21.
13. Fitzgerald GA, Patrono C. The coxibs, selective inhibitors of cyclooxygenase-2. N Engl J Med 2001;345:433-442.
14. Bombardier C, Laine L, Reicin A, Shapiro D, Burgos-Vargas R, Davis B, Day R, Ferraz MB, Hawkey CJ, Hochberg MC, Kvien TK, Schnitzer TJ; VIGOR Study Group. Comparison of upper gastrointestinal toxicity of Rofecoxib and naproxen in patients with rheumatoid arthritis. VIGOR Study Group. N Engl J Med 2000 Nov 23;343(21):1520-8.

ther nonsteroidal anti-inflammatory drugs: a 6-week and a 1-year trial in patients with osteoarthritis. Osteoarthritis Studies Group. Arch Fam Med 2000 Nov-Dec;9(10):1124-34.

1. Cannon et al. Rofecoxib, a specific inhibitor of cyclooxygenase 2, with clinical efficacity comparable with that diclofenac sodium. Arthritis&Rheum.200;43 (5):978-987.
2. Geusens PP, Truitt K, Sfikakis P, Zhao PL, DeTora L, Shingo S, Lau CS, Kalla A, Tate G.A A placebo and active comparator-controlled trial of Rofecoxib for the treatment of rheumatoid arthritis. Scand J Rheumatol 2002;31(4):230-8.
3. Chang DJ, Desjardins PJ, Chen E, Polis AB, McAvoy M, Mockoviak SH, Geba GP. Comparison of the analgesic efficacy of Rofecoxib and enteric-coated Diclofenac sodium in the treatment of postoperative dental pain: a randomized, placebo-controlled clinical trial. Clin Ther 2002 Apr;24(4):490-503.
4. Reicin A, Brown J, Jove M, deAndrade JR, Bourne M, Krupa D, Walters D, Seidenberg B.Efficacy of single-dose and multidose Rofecoxib in the treatment of post-orthopedic surgery pain. Am J Orthop 2001 Jan;30(1):40-8.
5. Lecomte J., Buyse H., Taymans J., Monti T. Treatment of tendinitis and bursitis: A comparison of Nimesulide and Naproxen Sodium in a double-blind parallel trial; Eur J Rheumatol Inflamm. 1994; 14(4):29-32.

**APPENDIX 1**

**ROFECOXIB LABELING**

**APPENDIX 2**

**DICLOFENAC LABELING**

**APPENDIX 3**

**JOBE'S TEST**

**Jobe's maneuver**:

The examiner stands in front of the patient who holds his/her arms in 90% abduction, 30% anterior flexion and medial rotation (thumbs towards the ground). The subject attempts to resist the lowering forces exerted by the examiner.

Resistance and pain: supraspinatus tendinitis

Pain and no resistance: supraspinatus tear

**
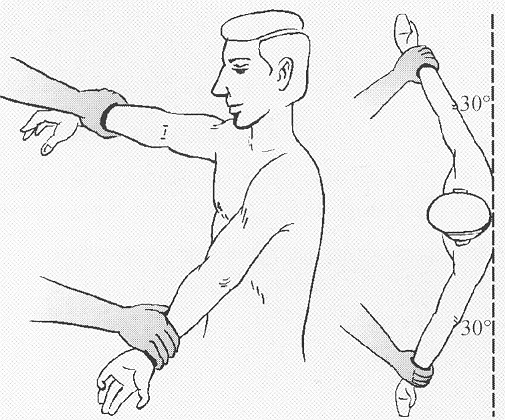
**

**APPENDIX 4**

**INFORMED CONSENT FORM**

**(strictly confidential)**

I, the undersigned: ....................................................., hereby agree to take part in the study 'Evaluation of the efficacy and safety of Rofecoxib in acute painful Rotator Cuff Syndrome'.

The objectives and conditions of the study in which I agree to take part have been explained to me by Dr. ............................................................

I have read and understood the patient information document given to me.

I have been free to ask all the questions I felt fit and to take into account the answers given to me.

I have clearly understood that my participation in the study is voluntary and that I can withdraw my consent at any time, irrespective of my reasons, without incurring any liability and without my so doing influencing the quality of the healthcare given to me or my physician's attention.

I agree to the documents in my medical file, which relate to the study, being accessed by the study managers and regulatory authorities. Other than those people who will process the information under the strictest medical secrecy, my anonymity will be protected.

I do not relinquish any of my statutory rights by signing this document,.

I agree to the data recorded during the study undergoing computerized processing by the sponsor or on his behalf. I have carefully noted that my right of access and rectification provided for under the 'Data processing and Freedom Act' (article 40) may be exercised at any time through Dr. ...............................................

Signed in ............................. on........./.........../.....................

**APPENDIX 5**

**PATIENT INFORMATION**

Dear Sir, Madam,

The aim of this form is to explain the study to you before you decide whether or not to take part.

Your physician has asked you to take part in the study entitled “Assessment of the efficacy and safety of rofecoxib in acute painful Rotator Cuff Syndrome”. The study is sponsored by Merck Sharp & Dohme-Chibret. All the information, which will be of use in helping you to decide whether or not to take part in the study, is given below. Before taking your decision, take the time to read this information document carefully and ask your physician any questions you think fit.

It is up to you to decide whether or not you wish to take part in the study. Your participation is entirely free and voluntary. You will certify your agreement to participate by signing the informed consent form of which you will keep a copy in compliance with Law No. 88-1138 dated December 20, 1988, known as the amended “Huriet Law”.

If you wish to ask a question or you experience a problem regarding the study, you can contact your investigating physician, Dr. ....................................., head of the study.

**STUDY OBJECTIVE**

The primary objective of the study is to assess the efficacy of rofecoxib in patients with Rotator Cuff Tendinitis (muscular structure of the shoulder), which is the condition from which you currently suffer. Rofecoxib is a medication under development by Laboratoires Merck Sharp & Dohme-Chibret. The medication has already shown its efficacy in numerous rheumatological fields (osteoarthritis, rheumatoid arthritis, etc.) but has never been evaluated in the context of shoulder tendon diseases. The aim of this study is to assess the efficacy of rofecoxib in that indication relative to a placebo and another anti-inflammatory medication: diclofenac.

In France, 270 patients like you are scheduled to take part in the study.

**STUDY MEDICATION**

Rofecoxib is a new medication from Laboratoires Merck Sharp & Dohme-Chibret. Rofecoxib is a member of the non-steroidal anti-inflammatory class of medications. Rofecoxib is a selective cyclooxygenase 2 inhibitor, which has already obtained regulatory approval from the Agence Française de Sécurité Sanitaire des Produits de Santé (AFSSAPS). During the clinical trials, the medication was shown to be effective on the symptoms of osteoarthritis and rheumatoid arthritis, particularly pain, with an efficacy comparable to that of other non-steroidal anti-inflammatories at high doses and with a superior safety profile. The patients receiving rofecoxib presented with significantly less serious gastrointestinal complications (perforation, ulcer and bleeding) and significantly less gastrointestinal adverse effects such as nausea, vomiting, acid reflux, digestion difficulties, and stomach discomfort, compared to the comparator medications.

**CONDITIONS OF STUDY PARTICIPATION**

You cannot take part in the study if one of the following applies to you:

- You are aged more than 60 years
- You have taken anti-inflammatories in the last 72 hours and/or acetaminophen in the last 12 hours and/or you have received a corticosteroid injection in the shoulder in the last 90 days.
- You suffer from a disease giving rise to inflammation of the joints.
- You suffer from active peptic ulcer or have experienced gastrointestinal bleeding.
- You are pregnant, or nursing.
- You have a history of inflammatory bowel disease
- You are know as having of impaired renal function.
- You have severe congestive heart failure
- You are known as having a moderate to severe liver failure.
- You are allergic to aspirin, diclofenac, other anti-inflammatories or selective cyclooxygenase 2 inhibitors or you show signs of hypersensitivity (asthma, acute rhinitis, nasal polyps, angioedema or urticaria).

Your participation in the study may be refused for other reasons that the study physician will explain to you.

Rofecoxib may interfere with other medications. It is therefore of the greatest importance to report all the treatments you are taking, including those not on medical prescription, to the study physician.

**DESCRIPTION OF THE STUDY**

The physician responsible for the study, Dr......................................, will question you on your medical history and examine you.

You will have one chance in three of receiving the study medication (rofecoxib), the comparator treatment (diclofenac) or a dummy devoid of pharmacological activity (placebo).

You can use symptomatic analgesic treatment for your shoulder tendinitis (acetaminophen/paracetamol at a maximum dosage of 4000 mg per day) as you require.

Throughout the study, each day, you will take 4 tablets: 1 tablet from bottle A every morning and 1 tablet from bottle B three times a day (morning, noon and night) at about the same time every day. Neither you nor your physician will know what treatment you are taking. In the event of an emergency, the identity of the treatment taken may be rapidly obtained.

The study will last one week. You will be asked to consult your physician twice:

- Today, when your physician will check that you fulfill the conditions required to take part in the study and, if that is the case, give you one of the treatments described above.

- One week later, for the second visit, in order to assess the efficacy of the treatment on your shoulder disease.

You will be asked to fill out a self-assessment diary twice a day (morning and night at regular times) for one week. The diary will assess the intensity of the pain in your shoulder during physical activities over the previous 12 hours (nighttime measurement) and over the preceding night (morning determination).

**EXPECTED BENEFITS FOR THE PATIENT**

The symptomatic treatment of shoulder tendinitis is based on analgesics and non-steroidal anti-inflammatories. The safety of rofecoxib, particularly at gastrointestinal level, has been shown to be favorable, compared to that of conventional anti-inflammatories.

The patients included in the study will be free to use analgesic symptomatic treatment (acetaminophen) at a maximum dosage of 4000 mg per day.

Two patients out of three will also receive symptomatic treatment of the shoulder tendinitis.

The patients included will, in principle, procure direct individual benefit. However, the treatment may prove ineffective and the patients will then procure no benefit. In all cases, your participation in the study will allow you to obtain regular monitoring of your disease.

**ADVERSE EFFECTS AND FORESEEABLE RISKS**

As with all medicinal products, rofecoxib is liable to induce adverse effects. Those most frequently reported in the clinical trials (frequency of 1 to 10%) were: abdominal pain, heartburn or stomach discomfort, diarrhea, nausea, digestive difficulties, edema, hypertension, dizziness, headache and itching.

The following occurred less frequently (0.1 to 1%): tiredness, abdominal distension, chest pain, constipation, oral ulceration, vomiting, gas, acid reflux, tinnitus, weight gain, muscle cramps, drowsiness, vertigo, depression, impaired intellectual capabilities, respiratory difficulties, redness, inflammation of the skin.

Rarely, moderate allergic reactions have been reported.

Similar adverse effects have been reported with diclofenac, together with allergic reactions, severe cutaneous reactions, gastrointestinal bleeding (spiting of blood or blood in the stools, black-colored stools).

**CONFIDENTIAL INFORMATION**

The information on you supplied for the study will be processed under the strictest conditions of confidentiality. You are free to withdraw from the study at any time without your so doing in any way changing your relationship with your physician. You should simply inform your physician and we request that you simply explain whether your decision is related to one of the study medications. You will then be offered another treatment.

**LEGAL ASPECT**

In compliance with the law dated December 20, 1988 (Huriet Law), this protocol was presented to the Institutional Review Board (CCPPRB) of PARIS COCHIN, which formulated a favorable opinion on its implementation on XXXXX

Laboratoires Merck Sharp & Dohme-Chibret have taken out insurance coverage for their third party liability in the context of this study. In addition, pursuant to the provisions of article R2038 of the Code of Public Health, the study medications will be supplied to you free of charge and the additional expenses relate to the specific visits required by the protocol will be assumed by Laboratoires Merck Sharp & Dohme-Chibret throughout the duration of the study.

After signing the informed consent form, you will continue to retain all your statutory rights. You have renounced none of your statutory rights in the event of negligence or professional misconduct by any person involved in the study.

Make sure that you have read and understood this document and that you have obtained answers to your questions before signing the informed consent form. This information document together with a copy of the consent form signed by you and your physician will be given to you.

**APPENDIX 6**

**INSURANCE CERTIFICATION**
